# Inhibition of Tumor Lipogenesis and Growth by Peptide‐Based Targeting of SREBP Activation

**DOI:** 10.1002/advs.202508111

**Published:** 2025-09-17

**Authors:** Shudi Luo, Huang Yang, Xiaoming Jiang, Zheng Wang, Xuxiao He, Ying Meng, Shan Li, Min Li, Daqian Xu, Zhengwei Mao, Zhimin Lu

**Affiliations:** ^1^ Zhejiang Key Laboratory of Pancreatic Disease Department of Gastroenterology The First Affiliated Hospital Zhejiang Key Laboratory of Frontier Medical Research on Cancer Metabolism Institute of Translational Medicine Zhejiang University School of Medicine Hangzhou Zhejiang 310029 China; ^2^ Institute of Fundamental and Transdisciplinary Research Cancer Center Zhejiang University Hangzhou Zhejiang 310029 China; ^3^ MOE Key Laboratory of Macromolecular Synthesis and Functionalization Department of Polymer Science and Engineering Zhejiang University Hangzhou 310027 China

**Keywords:** fatty acid synthesis, Insig1/2, LNP, PCK1, peptide, SREBP1, tumorigenesis

## Abstract

Tumor cells have substantially increased lipid biogenesis, which is primarily regulated by the activation of sterol regulatory element‐binding protein (SREBP). However, whether SREBP regulation can be targeted for cancer treatment remains unclear. Here, it is demonstrated that treating tumor cells with a peptide that replicates the amino acid sequence in Insig1/2 loop 1, which is the region where Insig1/2 interacts with AKT‐phosphorylated phosphoenolpyruvate carboxykinase 1 (PCK1), inhibits the IGF1‐induced interaction between PCK1 and Insig1/2. Consequently, this treatment abrogates PCK1‐mediated phosphorylation of Insig1 at S207 and Insig2 at S151, reduces the nuclear accumulation of SREBP1, and decreases SREBP1 activity‐dependent expression of lipid synthesis genes, lipid accumulation, and tumor cell proliferation. Intravenous administration of engineered liposomal nanoparticles (LNP)‐encapsulated Insig1/2 loop 1 peptide effectively suppresses tumor growth and extends mouse survival without apparent adverse effects. The peptide treatment, when combined with the receptor tyrosine kinase inhibitor lenvatinib or the anti‐obesity medication semaglutide, results in additive tumor inhibition. These findings highlight the potential of LNP‐Insig1/2 loop 1 peptide administration to inhibit SREBP activity—traditionally regarded as untargetable—for the treatment of human cancer.

## Graphical abstract

A peptide that replicates the amino acid sequence in Insig1/2 loop 1 inhibits the IGF1‐induced interaction between PCK1 and Insig1/2, PCK1‐mediated phosphorylation of Insig1/2, and SREBP activity‐dependent lipid synthesis. Administration of the LNP‐Insig1/2 loop 1 peptide effectively attenuates tumor growth and sensitizes tumors to the antitumor effect of lenvatinib or semaglutide

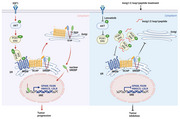

## 1 Introduction

De novo synthesis of lipids, including cholesterol and fatty acids, is critical for membrane biosynthesis, energy storage, and the generation of signaling molecules. This process is highly activated in tumor cells and supports tumor development.^[^ ^1^, ^2^ ^]^ Membrane‐bound transcription factor sterol regulatory element‐binding proteins (SREBPs), including the SREBP‐1a, SREBP‐1c/ADD1, and SREBP‐2 isoforms, regulate the transcription of genes required for lipid biosynthesis and cholesterol uptake.^[^ ^3^ ^]^ The function of SREBPs is regulated by endoplasmic reticulum (ER) anchor proteins, insulin‐induced genes (Insigs), and an escort protein, the SREBP cleavage‐activating protein SCAP.^[^ ^3^, ^4^, ^5^ ^]^ Two Insig isoforms that share 69% amino acid identity, Insig1 and Insig2, contain six transmembrane‐spanning regions and differ in their cytosolic N‐termini.^[^ ^6^, ^7^ ^]^ Insig proteins bind to oxysterols in the central cavities within their transmembrane domains, which is crucial for Insigs to bind to SCAP via transmembrane domains 3 and 4.^[^ ^3^, ^8^, ^9^, ^10^ ^]^ Insig inhibits the ER‐to‐Golgi transport of the SREBP–SCAP complex and SREBP activation and promotes the degradation of 3‐hydroxy‐3‐methylglutaryl (HMG) CoA reductase, thereby reducing cholesterol synthesis.^[^ ^1^ ^]^

Under low sterol conditions, which disrupt the Insig‐SCAP interaction, COPII mediates SCAP‐SREBP complex transport from the ER to the Golgi apparatus,^[^ ^4^, ^11^ ^]^ where the SREBPs are cleaved by the S1P and S2P proteases to yield active amino‐terminal fragments that enter the nucleus for gene transcription.^[^ ^1^, ^12^ ^]^ The subsequently increased sterol content restores the Insig‐SCAP interaction, thereby inhibiting SREBP activation and forming a feedback loop.^[^ ^3^, ^13^, ^14^ ^]^ However, in tumor cells, oncogenic signaling activates SREBP independent of the intracellular sterol level. Insulin‐like growth factor 1 (IGF1) stimulation induces AKT‐mediated phosphorylation of phosphoenolpyruvate carboxykinase 1 (PCK1) at S90. Phosphorylated PCK1, functioning as a protein kinase, binds to the loop1 region of Insig1/2 and phosphorylates Insig1 at S207 and Insig2 at S151. This phosphorylation reduces sterol binding to Insig1/2, thereby activating SREBP1/2 and promoting SREBP1/2‐mediated transcription of lipogenic genes that support tumor development.^[^ ^15^, ^16^ ^]^ Transcription factors, including SREBP, have long been considered untargetable for cancer treatment. The therapeutic potential of disrupting PCK1‐mediated SREBP activation remains unclear.

In this report, we demonstrated that a peptide that replicates the amino acid sequence in Insig1/2 loop 1 inhibits the IGF1‐induced interaction between PCK1 and Insig1/2, PCK1‐mediated phosphorylation of Insig1/2, and SREBP activity‐dependent lipid synthesis. Administration of the LNP‐Insig1/2 loop 1 peptide effectively attenuated tumor growth and sensitized tumors to the antitumor effect of lenvatinib or semaglutide.

## 2 Results

### 2.1 The Insig1/2 Loop 1 Peptide Binds to S90‐Phosphorylated PCK1 and Blocks PCK1‐Mediated SREBP Activation and Tumor Cell Proliferation

**Figure 1:**
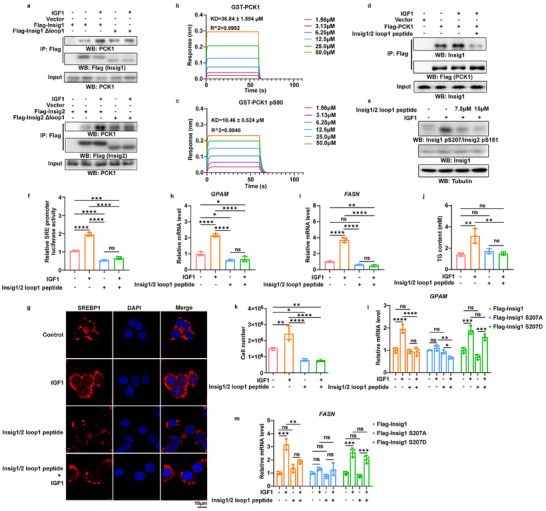
The Insig1/2 loop 1 peptide binds to S90‐phosphorylated PCK1 and blocks PCK1‐mediated SREBP activation and tumor cell proliferation. Immunoblotting analyses were performed using the indicated antibodies; representative results from three independent experiments are shown (IP, immunoprecipitation; WB, western blot). a) Huh7 cells expressing various Insig1 (top) or Insig2 (bottom) truncation mutants were treated with or without IGF1 (100 ng mL^−1^) for 1 h. b,c) Biolayer interferometry assays (Sartorius Octet) were employed to measure the binding affinity of Insig1/2 loop 1 peptide to GST‐PCK1 or GST‐PCK1 pS90 proteins. Data represent three independent experiments. d) Flag‐PCK1‐expressing Huh7 cells were pretreated with Insig1/2 loop 1 peptide (15 µm) for 1 h before IGF1 (100 ng mL^−1^) stimulation. e) Huh7 cells were pretreated with low (7.5 µm) or high (15 µm) concentrations of Insig1/2 loop 1 peptide for 1 h before IGF1 (100 ng mL^−1^) stimulation. f) Huh7 cells transiently transfected with β‐galactosidase and SRE‐driven luciferase reporter vectors were pretreated with Insig1/2 loop 1 peptide (15 µM) for 1 h, followed by stimulation with or without IGF1 (100 ng mL^−1^) for 14 h. SRE luciferase activity was normalized to β‐galactosidase activity. Data represent the mean ± SD (n  =  3). Statistical significance was determined by one‐way analysis of variance (ANOVA). g) Following pretreatment with Insig1/2 loop 1 peptide (15 µm) and IGF1 stimulation (100 ng mL^−1^, 8 h), Huh7 cells were subjected to immunofluorescence analysis (scale bar: 10 µm). h) Quantitative PCR analysis of SREBP1 target gene *GPAM* in Huh7 cells pretreated with Insig1/2 loop 1 peptide (15 µm) and stimulated with IGF1 (100 ng mL^−1^) for 10 h. Data represent the mean ± SD (n  =  3). Statistical significance was determined by one‐way analysis of variance (ANOVA). i) Quantitative PCR analysis of SREBP1 target gene *FASN* in Huh7 cells pretreated with Insig1/2 loop 1 peptide (15 µm) and stimulated with IGF1 (100 ng mL^−1^) for 10 h. Data represent the mean ± SD (n  =  3). Statistical significance was determined by one‐way analysis of variance (ANOVA). j) Intracellular triglyceride (TG) content was quantified in Huh7 cells pretreated with Insig1/2 loop 1 peptide (25 µm) and stimulated with IGF1 (100 ng mL^−1^) for 24 h. Data represent the mean ± SD (n  =  3). Statistical significance was determined by one‐way analysis of variance (ANOVA). k) Cell proliferation was assessed by automated cell counting after 72 h of IGF1 stimulation (100 ng mL^−1^) in Huh7 cells pretreated with Insig1/2 loop 1 peptide (25 µm) for 1 h. Data represent the mean ± SD (n  =  3). Statistical significance was determined by one‐way analysis of variance (ANOVA). l) FLAG‐Insig1, Flag‐Insig1 S207A or FLAG‐ Insig1 S207D was expressed in Huh7 cells with depletion of Insig1 by expressing Insig1 shRNA. Quantitative PCR analysis of SREBP1 target gene *GPAM* in Huh7 cells pretreated with Insig1/2 loop 1 peptide (15 µm) and stimulated with IGF1 (100 ng mL^−1^) for 10 h. Data represent the mean ± SD (n  =  3). Statistical significance was determined by two‐way analysis of variance (ANOVA). m) FLAG‐Insig1, Flag‐Insig1 S207A or FLAG‐ Insig1 S207D was expressed in Huh7 cells with depletion of Insig1 by expressing Insig1 shRNA. Quantitative PCR analysis of SREBP1 target gene *FASN* in Huh7 cells pretreated with Insig1/2 loop 1 peptide (15 µm) and stimulated with IGF1 (100 ng mL^−1^) for 10 h. Data represent the mean ± SD (n  =  3). Statistical significance was determined by two‐way analysis of variance (ANOVA). *p < 0.05, **p < 0.01, ***p < 0.001, ****p < 0.0001; ns, no significance.

The first loop of Insig1 and Insig2 has been suggested to be responsible for their interactions with PCK1 in 293T cells in an AKT‐dependent manner, specifically requiring PCK1 S90 phosphorylation.^[^ ^15^ ^]^ Consistently, the expression of Insig1 (**Figure** **1**a, top panel) and Insig2 (Figure 1a, bottom panel) mutants with deletion of the first loop inhibited IGF1 treatment‐enhanced binding of PCK1 to Insig1 and Insig2 in Huh7 human hepatocellular carcinoma (HCC) cells. We next synthesized an Insig1/2 loop 1 peptide (143‐LYPCIDSHLGEPHKFKRE‐160) and incubated it with purified bacteria‐expressed WT GST‐PCK1 (Figure S1a, Supporting Information) and S90‐phosphorylated GST‐PCK1 (Figure S1b, Supporting Information). Octet analyses revealed that the Insig1/2 loop 1 peptide exhibited much stronger binding affinity for the S90‐phosphorylated GST‐PCK1 protein than nonphosphorylated GST‐PCK1 (Figure 1b,c).

To determine whether the Insig1/2 loop 1 peptide regulates the interaction between PCK1 and Insig1/2 in tumor cells, we treated Huh7 and Hep3B HCC cells with this peptide fused with a fragment of transactivator of transcription (TAT) peptide to facilitate plasma membrane penetration,^[^ ^17^ ^]^ resulting in ≈100% tumor cells internalizing the peptide (Figure S1c, Supporting Information). The Insig1/2 loop 1 peptide markedly inhibited IGF1‐induced binding of PCK1 to Insig1 (Figure 1d; Figure S1d, Supporting Information), Insig1 S207/Insig2 S151 phosphorylation (Figure 1e; Figure S1e, Supporting Information), SREBP1 activity (Figure 1f), the nuclear accumulation of SREBP1 (Figure 1g), and the expression of *glycerol‐3‐phosphate acyltransferase mitochondrial* (*GPAM*) (Figure 1h) and *fatty acid synthase* (*FASN*) (Figure 1i), which are regulated by SREBP1 and play central roles in de novo lipogenesis.^[^ ^12^ ^]^ In addition, treatment of HCC cells with the peptide inhibited IGF1‐induced nuclear translocation of SREBP2 (Figure S2a, Supporting Information) and suppressed SREBP2‐mediated expression of *3‐hydroxy‐3‐methylglutaryl‐CoA synthase 1* (*HMGCS1*) (Figure S2b, Supporting Information) and the cholesterol transporter ***l*** *ow‐density lipoprotein (LDL) receptor* (*LDLR*) (Figure S2c, Supporting Information). As expected, the Insig1/2 loop 1 peptide reduced the IGF1‐induced increase in triglycerides (TGs) (Figure 1j; Figure S1f, Supporting Information) and inhibited the IGF1‐induced increase in the proliferation of Huh7 and Hep3B cells (Figure 1k; Figure S1g, Supporting Information). Furthermore, peptide treatment inhibited Insig1 S207 phosphorylation (Figure S1h, Supporting Information) and induced cell death in HCT116 human colon cancer cells. (Figure S1i, Supporting Information). Notably, expression of the phosphorylation‐mimicking Insig1 S207D mutant in Huh7 cells abrogated the peptide‐mediated suppression of nuclear translocation of SREBP1 (Figure S2d,e, Supporting Information) and expression of *GPAM* (Figure 1l) and *FASN* (Figure 1m). These results indicate that the Insig1/2 loop 1 peptide binds to S90‐phosphorylated PCK1 and blocks PCK1‐mediated SREBP activation, SREBP‐downstream gene expression, de novo lipogenesis, and tumor cell proliferation.

### 2.2 LNPs with a 1:1 Ratio of TAT to cRGD Effectively Accumulate in Tumor Tissues

**Figure 2:**
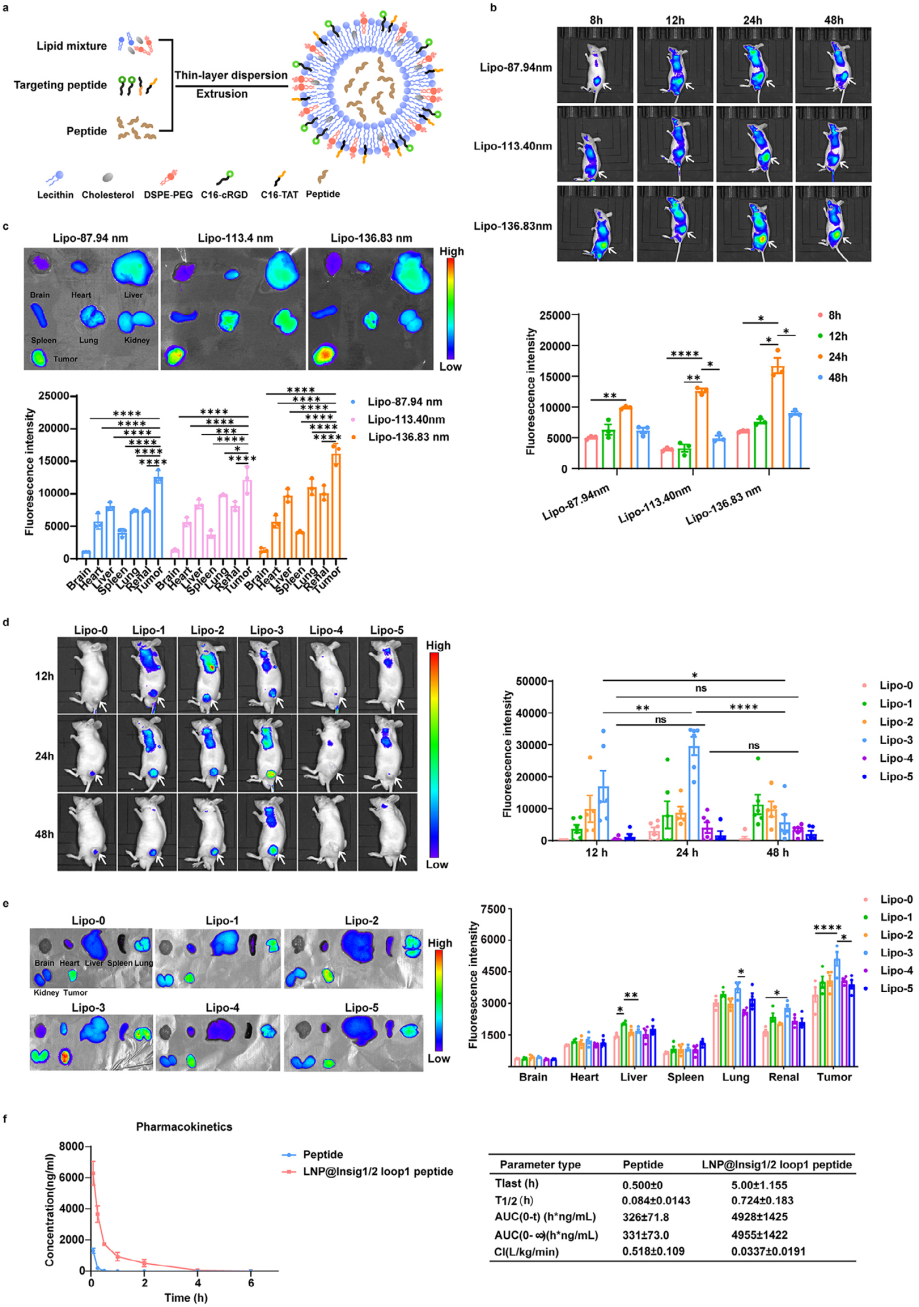
LNPs with a 1:1 ratio of TAT to cRGD effectively accumulate in tumor tissues. a) Schematic illustration of LNP composition. b,c) Biodistribution analysis of differently sized LNPs in Huh7 tumor‐bearing BALB/c‐nude mice (n = 3). (b) In vivo fluorescence imaging at 8, 12, 24, and 48 h post‐intravenous injection (10 mg kg^−1^). The arrows indicate the tumor site. (c) Ex vivo fluorescence imaging of tumors and major organs harvested 48 h post‐injection. d,e) Optimization of TAT/cRGD peptide coating ratios for tumor‐targeted LNPs (n = 3). (d) In vivo fluorescence imaging at 12, 24, and 48 h post‐injection. The arrows indicate the tumor site. (e) Ex vivo fluorescence imaging of tumors and organs at 48 h. f) Pharmacokinetic profile of LNP@Insig1/2 loop 1 peptide versus free peptide (Insig1/2 loop 1 peptide) following intravenous administration in ICR mice (n = 3, 10 mg kg^−1^). Plasma concentration‐time curves were quantified, and pharmacokinetic parameters were calculated: AUC (0–t) (h·ng mL^−1^) and AUC (0–∞) (h·ng mL^−1^). Data are expressed as mean ± SD (n  =  3). Statistical significance was determined by one‐way analysis of variance (ANOVA). * *p* < 0.05, ** *p* < 0.01, *** *p* < 0.001, **** *p* < 0.0001, ns, no significance.

Liposomal nanoparticles (LNP)‐based delivery has emerged as a new type of therapeutic approach to prevent and treat various diseases.^[^ ^18^, ^19^, ^20^ ^]^ To examine the therapeutic potential of the Insig1/2 loop 1 peptide, we designed liposomes and encapsulated an IR750 dye with average sizes of approximately 87.94, 113.40, and 136.83 nm (**Figure** **2**a; Figure S3a–c, Supporting Information). We next subcutaneously injected Huh7 cells into nude mice. The intravenous injection of LNPs into mice revealed that LNPs that were 136.83 nm in size accumulated more in tumor tissues than LNPs that were 113.40 nm and 87.94 nm in size (Figure 2b). In addition, a time course injection of LNPs revealed that LNPs levels in tumor tissues peaked 24 h after injection. Analyses of mouse organs revealed some accumulation in the liver, kidney, and lung, with limited distribution in the brain, heart, and spleen (Figure 2c). These results indicate that LNPs with an average size of 136.83 nm effectively accumulate in tumor tissue.

The cyclic tripeptide Arg‐Gly‐Asp (cRGD), which binds to integrin receptors, has been shown to bind to tumor cells.^[^ ^17^ ^]^ Administration of non‐functional TAT variants or depletion of integrin αvβ3 substantially reduced peptide accumulation in tumor cells in vitro (Figure S3d, Supporting Information) and in mouse tumors in vivo (Figure S3f,g, Supporting Information). Intravenous injection of LNPs (136.83 nm) with different ratios of TAT and cRGD on the surface of LNPs (Figure S3e, Supporting Information) showed that LNPs with a 1:1 ratio (10% each with 20% total) on the LNP surface exhibited more accumulation in tumor tissues and lungs than the LNPs with no TAT/cRGD or with different ratios of TAT/cRGD (Figure 2d,e). Moreover, the LNPs maintained measurable accumulation in tumor tissues through 48 h.

Following the aforementioned material optimization, the Insig1/2 loop 1 peptide was encapsulated within LNPs with a size of 136.83 nm and a 1:1 ratio of TAT to cRGD, designated as LNP@Insig1/2 loop 1 peptide for subsequent experimental investigations. High‐performance liquid chromatography (HPLC) analysis revealed that the encapsulation efficiency (EE) and loading efficiency (LE) of LNP@Insig1/2 loop 1 peptide were 87.4% and 6.78%, respectively (Figure S3h–j, Supporting Information).

To evaluate the pharmacokinetic profile in vivo, either LNP@Insig1/2 loop 1 peptide or an equivalent dose of the free Insig1/2 loop 1 peptide was administered to mice via intravenous injection. Liquid chromatography–mass spectrometry (LC–MS) analysis of mouse serum revealed that the LNP@ Insig1/2 loop 1 peptide had a much longer half‐life (T1/2) (≈0.724 h) than did the Insig1/2 loop 1 peptide alone (≈0.084 h) (Figure 2f). The mean area under the curve (AUC) _(0‐t)_ of the LNP@Insig1/2 loop 1 peptide (4928 h*ng mL^−1^) was approximately 15.1‐fold greater than that of the Insig1/2 loop 1 peptide alone (326 h*ng mL^−1^). In addition, the LNP@Insig1/2 loop 1 peptide had a much lower clearance (CL) than the Insig1/2 loop 1 peptide alone (Figure 2f). These results suggest that LNPs significantly extend the half‐life of the peptide in blood and increase its bioavailability.

### 2.3 Insig1/2 Loop 1 Peptide Treatment Inhibits Tumor Growth and Promotes Lenvatinib‐Based HCC Treatment

**Figure 3:**
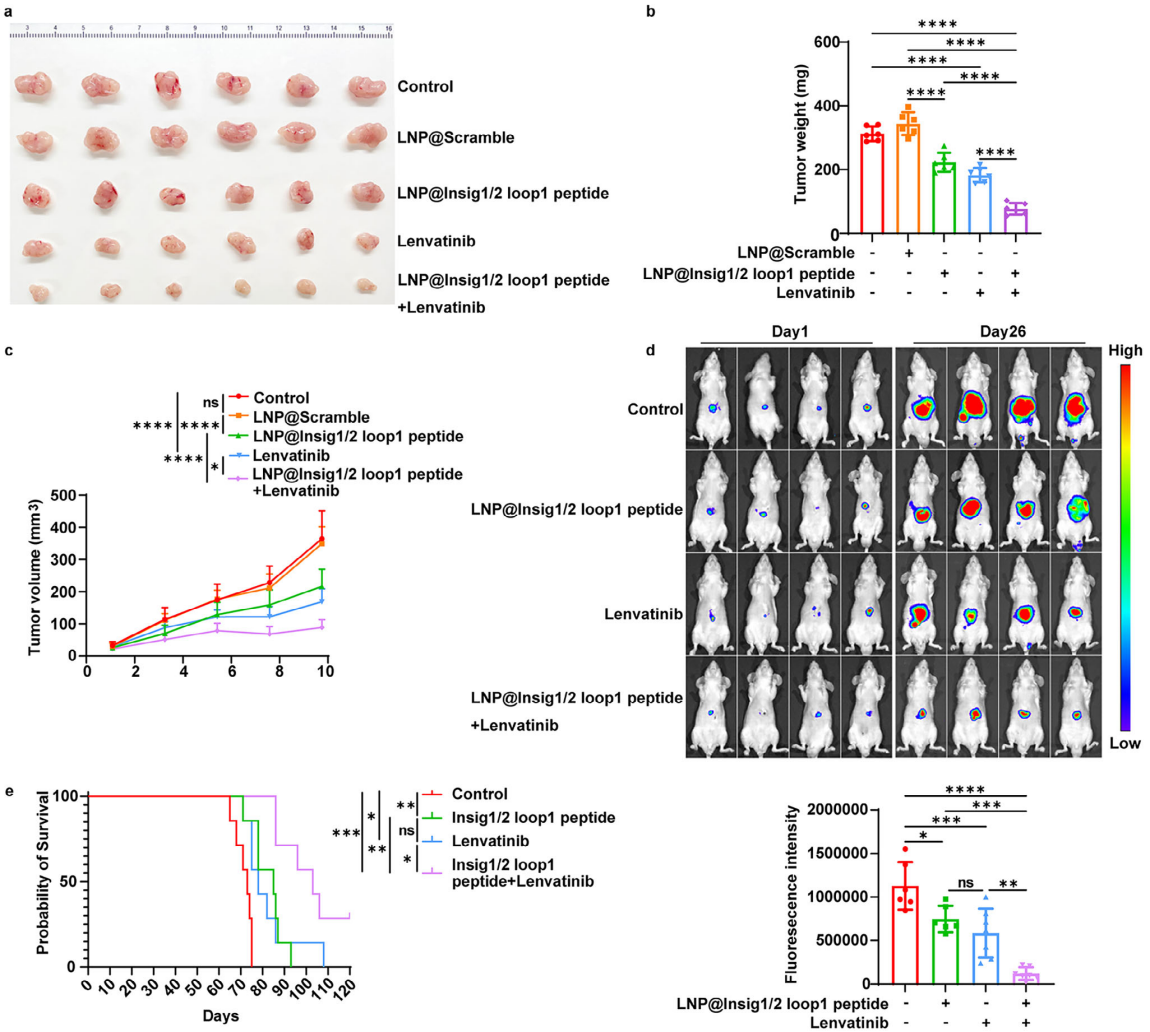
Insig1/2 loop 1 peptide treatment inhibits tumor growth and promotes lenvatinib‐based HCC treatment. a–c) Huh7 cells were subcutaneously implanted into nude mice (n = 6 per group). Mice received intravenous injections of either control LNP@scramble or LNP@Insig1/2 loop 1 peptide (10 mg kg^−1^, every other day), and/or daily oral administration of lenvatinib mesylate (2 mg kg^−1^). (a) Representative optical images of tumors, (b) tumor weights, and (c) tumor volume measurements are shown. Statistical significance was determined by one‐way analysis of variance (ANOVA). d,e) For the orthotopic model, Huh7 cells were intrahepatically injected into nude mice (n = 7 per group). Treatment groups received intravenous injections of LNP@Insig1/2 loop 1 peptide (10 mg kg^−1^, every other day) and/or oral administration of lenvatinib mesylate (2 mg kg^−1^, daily). (d) Tumor progression was monitored by in vivo imaging system, and (e) survival analysis was performed using Kaplan‐Meier curves. Statistical significance was determined by Log‐rank (Mantel‐Cox) test. Data are expressed as mean ± SD (b,d) or mean ± SEM (c). Statistical significance was determined by one‐way analysis of variance (ANOVA). *p < 0.05, ** *p* < 0.01, *** *p* < 0.001, **** *p* < 0.0001, ns, no significance.

To examine the effect of the Insig1/2 loop 1 peptide on tumor growth, we subcutaneously injected Huh7 cells into nude mice. The intravenous injection of LNPs (136.83 nm, 1:1 ratio of TAT and cRGD) encapsulating the Insig1/2 loop 1 peptide (LNP@Insig1/2 loop 1 peptide) or LNPs with a scramble peptide (LNP@scramble) into mice revealed that the LNPs with a scramble peptide had no obvious effect on tumor growth (**Figure** **3**a–c). In contrast, the Insig1/2 loop 1 peptide inhibited tumor growth (Figure 3a) and reduced tumor weight (Figure 3b) and volume (Figure 3c). In addition, Insig1/2 loop 1 peptide injection inhibited intrahepatically injected tumor cell growth in the liver (Figure 3d; Figure S4a, Supporting Information) and prolonged mouse survival (Figure 3e; Figure S4b, Supporting Information). These results indicate that the Insig1/2 loop 1 peptide impedes liver tumor growth in mice.

Lenvatinib, a tyrosine kinase inhibitor, serves as a first‐line treatment for patients with advanced HCC.^[^ ^21^ ^]^ However, it can induce treatment resistance through activation of bypass signaling pathways, including the EGFR and FGFR pathways.^[^ ^22^ ^]^ We next treated the mice harboring Huh7 cell‐derived HCC tumors with a combination of lenvatinib and the Insig1/2 loop 1 peptide, which exhibited an additive inhibition of tumor growth (Figure 3a–d) and prolonged mouse survival time (Figure 3e) compared with lenvatinib or the Insig1/2 loop 1 peptide alone. These results suggest that the Insig1/2 loop 1 peptide in combination with lenvatinib improves the current lenvatinib‐based HCC treatment.

### 2.4 Insig1/2 Loop 1 Peptide Treatment Inhibits SREBP1 Activity and Lipid Synthesis and Induces Tumor Cell Apoptosis in Liver Tumors

**Figure 4:**
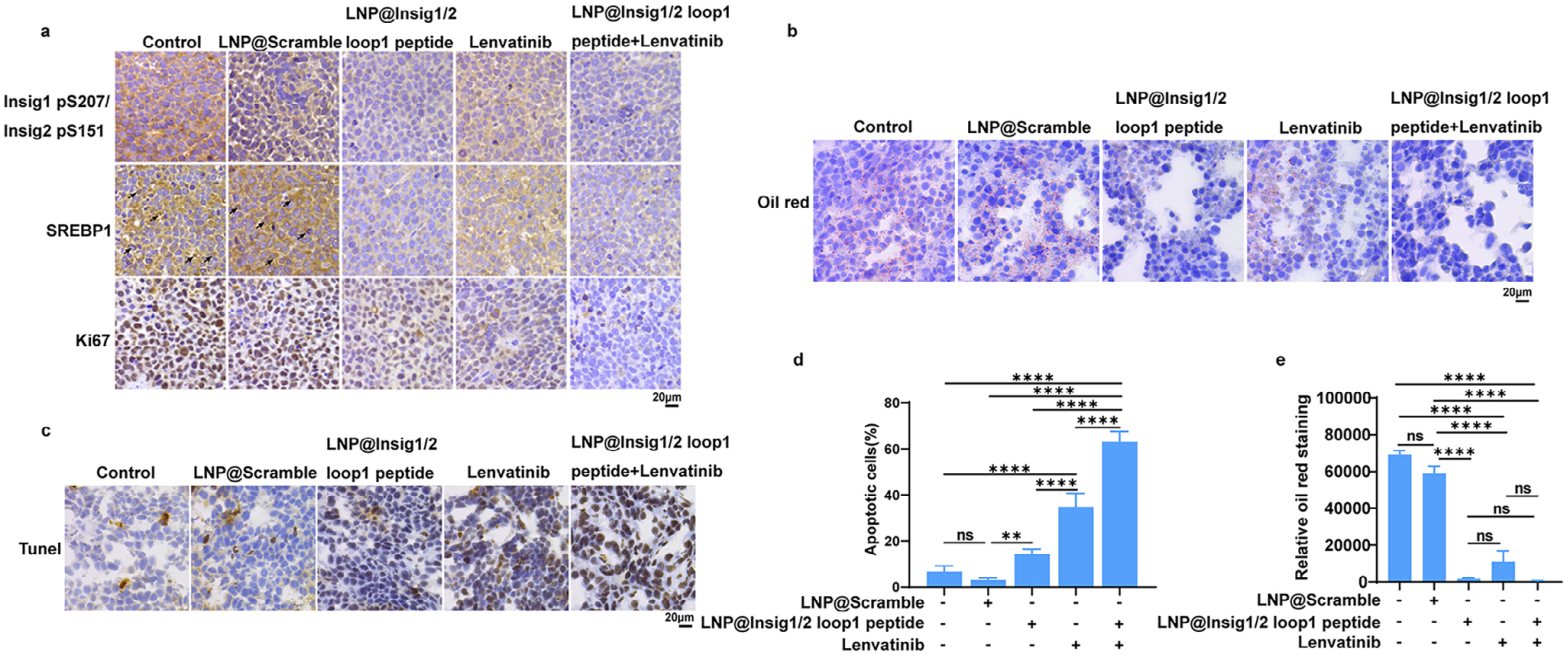
Insig1/2 loop 1 peptide treatment inhibits SREBP1 activity and lipid synthesis and induces tumor cell apoptosis in liver tumors. a) Immunohistochemical (IHC) analysis of mouse tumor specimens were performed using the indicated antibodies. Representative micrographs are shown (scale bar: 20 µm). b,c) Cryosections of mouse tumor tissues were prepared for histological analysis. (b) Lipid accumulation was assessed by Oil Red O staining. Representative micrographs are shown (scale bar: 20 µm). (c) Apoptotic cells were detected by TUNEL assay. Representative micrographs are shown (scale bar: 20 µm). d,e) Quantitative analysis of (d) apoptotic areas (TUNEL‐positive regions) and (e) lipid droplet counts (Oil Red O‐positive particles) was performed. Data represent the mean ± SD (n  =  4). Statistical significance was determined by one‐way analysis of variance (ANOVA). ** *p* < 0.01, **** *p* < 0.0001, ns, no significance.

To explore whether Insig1/2 loop 1 peptide treatment regulates SREBP activity in tumors, we performed immunohistochemical (IHC) analyses of mouse tumors derived from Huh7 cells. We showed that the Insig1/2 loop 1 peptide reduced Insig1 S207/Insig2 S151 phosphorylation, SREBP1 expression, and nuclear accumulation, and Ki67 expression (**Figure** **4**a). In addition, Oil Red O staining revealed that the Insig1/2 loop 1 peptide reduced lipid accumulation in tumor tissues (Figure 4b,e). As expected, these reductions were also observed following lenvatinib treatment (Figure 4a,b), which inhibited AKT activation.^[^ ^23^ ^]^ We next performed TUNEL analyses and showed that both the Insig1/2 loop 1 peptide and lenvatinib treatment induced cell apoptosis in tumor tissues (Figure 4c,d). Notably, an additive effect was observed after combined treatment with the Insig1/2 loop 1 peptide and lenvatinib. These results indicate that the Insig1/2 loop 1 peptide inhibits SREBP1 activity and lipid synthesis in tumor cells and induces tumor cell apoptosis in liver tumors.

Intravenous injection of the LNPs with the Insig1/2 loop 1 peptide into mice did not result in obvious adverse effects, as revealed by the results from hematoxylin and eosin (H&E) staining of the heart, liver, spleen, lung, and kidney tissues (Figure S4c, Supporting Information), and blood examination of the liver, heart, and kidney (Figure S4d–k, Supporting Information). In addition, analysis of normal mouse liver tissues revealed that treatment with LNP@Insig1/2 loop 1 peptide did not significantly alter the mRNA expression levels of lipid synthesis genes *FASN*, *GPAM*, and *HMGCS1*, or *LDLR* (Figure S4l–o, Supporting Information). These results indicate that the Insig1/2 loop 1 peptide has limited toxicity.

### 2.5 The Insig1/2 Loop 1 Peptide in Combination with Semaglutide Treatment Induces an Additive Tumor Inhibitory Effect

**Figure 5:**
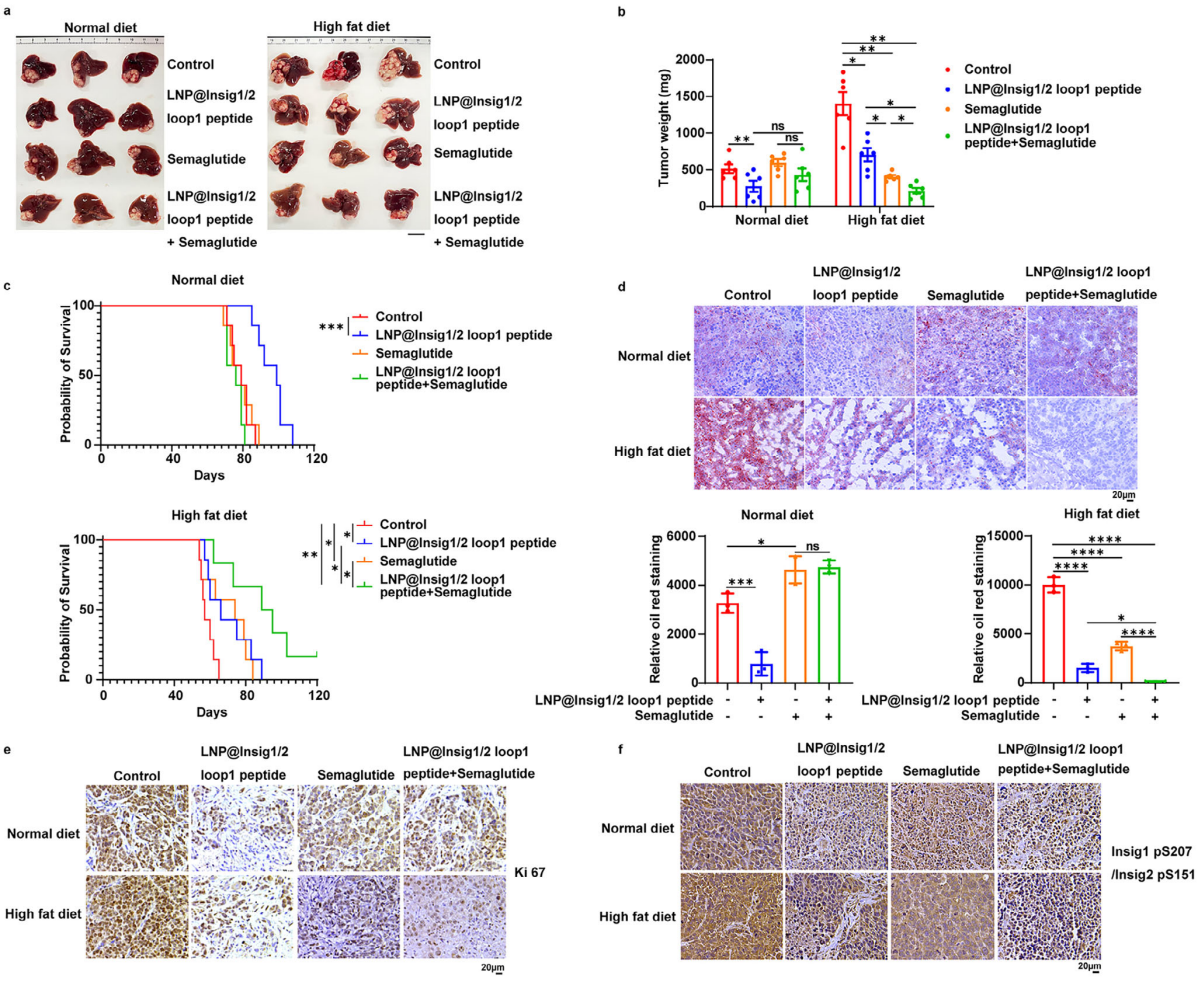
The Insig1/2 loop 1 peptide in combination with semaglutide treatment induces an additive tumor inhibitor effect. a–c) Huh7 cells were orthotopically injected into the liver of nude mice (n = 7 per group). Treatment groups received intravenous injections of LNP@Insig1/2 loop 1 peptide (10 mg kg^−1^, every other day), and/or intraperitoneal injection of Semaglutide (30 nmol kg^−1^, daily). (a) Representative optical images of tumors, (b) tumor weights, data are expressed as mean ± SD, statistical significance was determined by one‐way analysis of variance (ANOVA), and (c) survival analysis was performed using Kaplan‐Meier curves. Statistical significance was determined by Log‐rank (Mantel‐Cox) test. d) Cryosections of mouse tumor tissues were prepared, and lipid accumulation was assessed by Oil Red O staining. Representative micrographs are shown (scale bar: 20 µm). Quantitative analysis of lipid droplet counts (Oil Red O‐positive particles) was performed. Data are expressed as mean ± SD (n  =  3). Statistical significance was determined by one‐way analysis of variance (ANOVA). e) Immunohistochemical (IHC) analysis of mouse tumor specimens was performed using the Ki67 antibody. Representative micrographs are shown (scale bar: 20 µm). Data are expressed as mean ± SD (n  =  3). Statistical significance was determined by one‐way analysis of variance (ANOVA). f) Immunohistochemical (IHC) analysis of mouse tumor specimens was performed using the Insig1 pS207/Insig2 pS151 antibody. Representative micrographs are shown (scale bar: 20 µm). * *p* < 0.05, ** *p* < 0.01, *** *p* < 0.001, **** *p* < 0.0001, ns, no significance.

Tumor cell proliferation requires not only de novo lipid synthesis but also the uptake of lipids by tumor cells.^[^ ^24^, ^25^ ^]^ Semaglutide, which has been used as an anti‐obesity medication for long‐term weight management, has been shown to reduce the expression of enzymes for fatty acid metabolism.^[^ ^26^ ^]^ We orthotopically injected Huh7 cells into the mouse liver. Strikingly, treatment of the mice with semaglutide markedly inhibited high‐fat diet‐induced tumor growth (**Figure** **5**a,b) and prolonged the survival time of mice (Figure 5c). Notably, the combination of semaglutide and the Insig1/2 loop 1 peptide demonstrated additive antitumor effects in mice fed a high‐fat diet. (Figure 5a–c). IHC analyses and Oil Red O staining of tumor tissues revealed that semaglutide administration decreased high‐fat diet‐increased lipid levels (Figure 5d) and Ki67 expression in tumor tissues (Figure 5e), and this reduction was further promoted by combined treatment with the Insig1/2 loop 1 peptide. Notably, in line with a previous report showing that a high‐fat diet activates AKT and promotes metabolic dysfunction‐associated steatohepatitis (MASH) and liver tumorigenesis,^[^ ^27^ ^]^ the high‐fat diet enhanced Insig1 S207 and Insig2 S151 phosphorylation (Figure 5f). This enhancement was reduced by treatment with the Insig1/2 loop 1 peptide as well as semaglutide administration. The combined treatment resulted in a further reduction in this phosphorylation. Of note, semaglutide significantly reduced circulating low‐density lipoprotein cholesterol (LDL‐C) (Figure S5a, Supporting Information) and high‐density lipoprotein cholesterol (HDL‐C) levels (Figure S5b, Supporting Information), while it had no significant effect on AKT‐mediated Insig1 S207 phosphorylation (Figure S5d, Supporting Information) or lipid uptake (Figure S5c, Supporting Information) in cultured HCC cells. These results suggest that semaglutide, which does not directly regulate the PCK1–Insig phosphorylation axis, reduces tumor growth by lowering lipid availability in the tumor microenvironment. Together, these findings indicate that combining the Insig1/2 loop1 peptide with semaglutide treatment produces an additive tumor‐inhibitory effect.

## 3 Discussion

**Figure 6:**
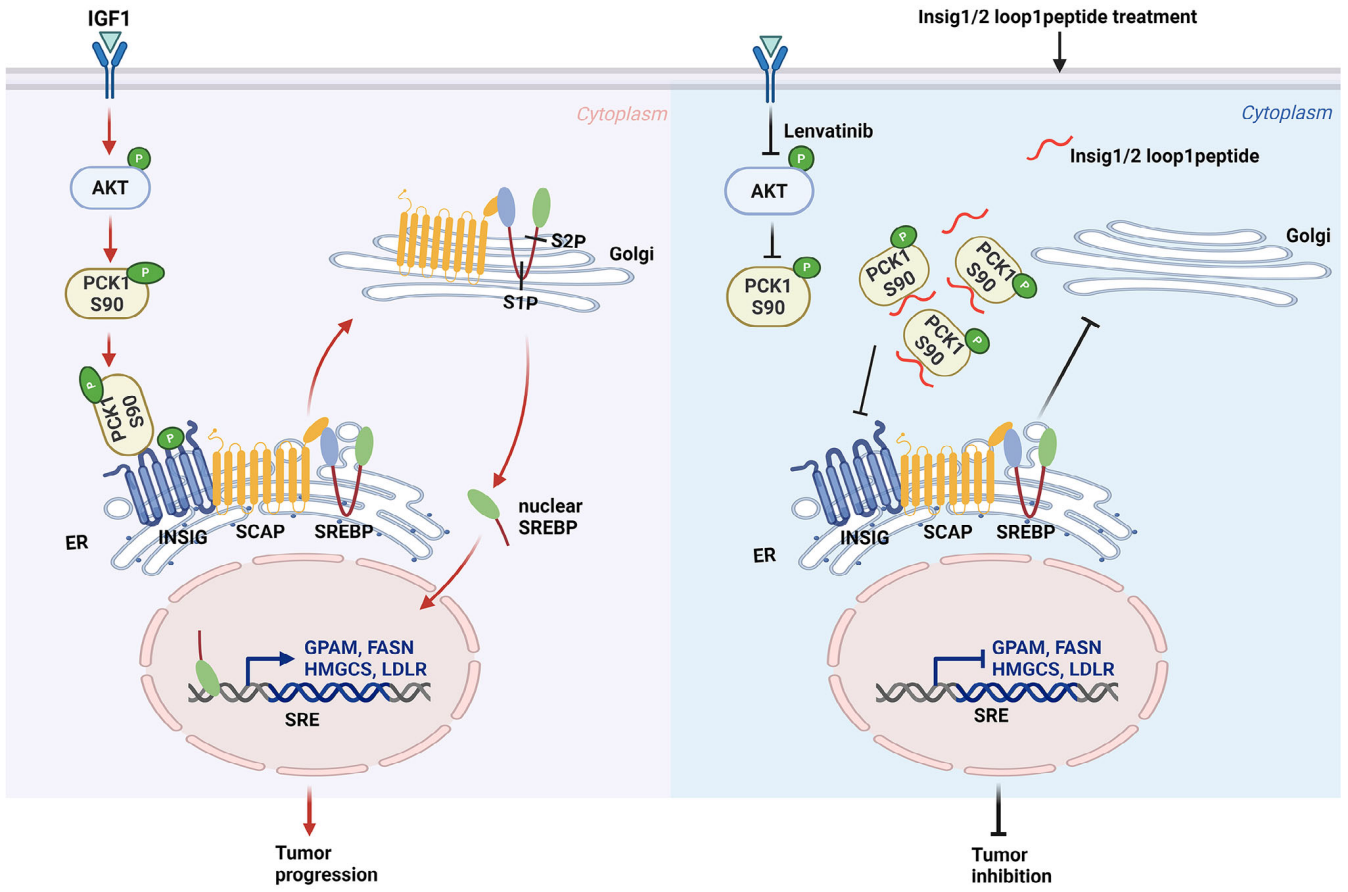
Mechanistic insights into tumor growth inhibition by Insig1/2 loop1 peptide through PCK1‐mediated SREBP1 activation and lipogenesis regulation. Schematic diagram of the peptide‐mediated tumor proliferation inhibition mechanism. Created in https://BioRender.com.

Tumor cells exhibit increased lipid biogenesis to sustain rapid growth and proliferation. The transcription factor SREBP is crucial for the expression of genes involved in the synthesis of fatty acids and cholesterol. In normal cells, SREBP activity is regulated by intracellular sterol levels through a feedback mechanism, leading to SREBP downregulation under high sterol conditions. However, in tumor cells, SREBP remains activated even in the presence of high sterol levels. This process is regulated by AKT‐mediated phosphorylation of PCK1 at S90, which promotes the interaction between PCK1 and Insig, followed by Insig phosphorylation.^[^ ^15^ ^]^ To explore the therapeutic potential of disrupting the PCK1‐Insig‐SREBP axis, we designed a peptide that replicates the amino acid sequence of Insig1/2 loop 1, the region that interacts with PCK1. Treatment of HCC cells with the Insig1/2 loop 1 peptide disrupted the IGF1‐induced PCK1‐Insig1/2 interaction, PCK1‐mediated phosphorylation of Insig1 at S207 and Insig2 at S151, nuclear accumulation of SREBP1, SREBP1 activity, downstream lipid synthesis gene expression, lipid accumulation, and tumor cell proliferation. Intravenous delivery of the Insig1/2 loop 1 peptide effectively attenuated tumor growth, prolonged mouse survival, inhibited the PCK1‐Insig‐SREBP‐lipid production axis, and increased cell apoptosis in tumor tissues. In addition, combination treatment with lenvatinib and the Insig1/2 loop 1 peptide exhibited an additive tumor‐inhibitory effect (**Figure** **6**).

LNP‐based delivery has been increasingly and intensively used for preventing and treating various diseases.^[^ ^18^, ^19^, ^20^ ^]^ Composed of either natural or synthetic lipids, LNPs have been used as versatile delivery systems for bioactive compounds, exhibiting favorable properties such as biocompatibility, biodegradability, low toxicity, and non‐immunogenicity.^[^ ^28^ ^]^ LNPs have been clinically used as effective delivery systems for some drugs, such as paclitaxel, doxorubicin, and methotrexate, to increase their effectiveness.^[^ ^29^ ^]^ The LNPs, with a diameter of 136.83 nm and a 1:1 ratio of TAT to cRGD, exhibited enriched accumulation in tumor tissues. In addition, the LNPs encapsulating the Insig1/2 loop 1 peptide prolonged the peptide's half‐life in mouse serum compared with that of the naked form, indicating that LNP‐based delivery can overcome the rapid turnover disadvantage of naked peptide delivery. Although the LNPs containing the peptide also accumulated in organs such as the lungs, liver, and kidneys, blood tests and structural and functional examinations of these organs did not reveal any obvious adverse effects. These findings underscore the potential of LNP‐Insig1/2 loop 1 peptide administration to inhibit SREBP activity.

Current inhibitors targeting the Insig1/2/SCAP/SREBP complex include Fatostatin and PF‐429242. Fatostatin binds to SCAP to inhibit SREBP1 translocation from the endoplasmic reticulum to the Golgi apparatus, thereby blocking SREBP activation.^[^ ^30^ ^]^ PF‐429242 suppresses the proteolytic activity of site‐1 protease (S1P), preventing the cleavage‐mediated activation of both SREBP1 and SREBP2 in the Golgi compartment.^[^ ^31^, ^32^ ^]^ Both Fatostatin and PF‐429242 are small molecules that inhibit SREBP activation under both physiological and pathological conditions, which may lead to undesired adverse effects. In contrast, our peptide‐based approach targets the PCK1/Insig protein‐protein interaction with high specificity and selectivity, with no observed side effects. Importantly, our strategy selectively inhibits hyperactivated SREBP induced by oncogenic signals while preserving physiological lipid metabolism. By employing engineered LNPs, we markedly extended the peptide's half‐life, improved its targeting specificity, and minimized off‐target effects.

Semaglutide has been used as an anti‐obesity medication for long‐term weight management and for the treatment of type 2 diabetes patients.^[^ ^33^, ^34^ ^]^ However, whether semaglutide has any therapeutic effect on human cancer remains unclear. We showed that semaglutide impeded high‐fat‐promoted tumor progression. Importantly, the reduction in lipid levels in tumors by semaglutide combined with the inhibition of de novo lipid synthesis via the Insig1/2 loop 1 peptide produced an additive tumor‐inhibiting effect and prolonged mouse survival. These findings provide an unprecedented strategy for treating human cancer by inhibiting lipid metabolism in cancer cells through a combined approach.

## 4 Experimental Section

### Materials

Rabbit polyclonal antibodies against Insig1 pS207/Insig2 pS151 and PCK1 pS90 were obtained from Signalway Biotechnology (Pearland, TX). PCK1 (A2036) antibodies were purchased from ABclonal, while INSIG1 (A‐9) (sc‐390504) antibodies were acquired from Santa Cruz Biotechnology (Dallas, TX). For immunofluorescence and IHC analyses, SREBP1 (NB100‐2215) and SREBP2 (NB100‐74543) antibodies were sourced from Novus. Recombinant IGF1 (HY‐P7018) was purchased from MedChemExpress. Mouse monoclonal anti‐Flag (F1804) and Flag M2 magnetic beads (M8823) were obtained from Sigma‐Aldrich. Alexa Fluor 555‐conjugated goat anti‐rabbit secondary antibody (A21428) and BODIPY FL C16 (D3821) were procured from Thermo Fisher Scientific (Waltham, MA). Oil Red O (C0157S), TUNEL staining kits (C1098), and the Amplex Red Triglyceride Assay Kit (S0219S) were supplied by Beyotime Biotechnology (Jiangsu, China). All custom peptides were synthesized by Nanjing Yuanpeptide Biotechnology Co., Ltd. (Nanjing, Jiangsu). Insig1/2 loop1 peptide sequence, RKKRRQRRRLYPCIDSHLGEPHKFKRE; scramble peptide sequence, RKKRRQRRRRCPHFEDYKEGPLSLKIH; Biotin‐peptide‐TAT, Biotin‐LYPCIDSHLGEPHKFKRERKKRRQRRR; Biotin‐peptide‐TAT LOF, Biotin‐LYPCIDSHLGEPHKFKREAAEAAQAAE.

### DNA Constructs and Mutagenesis

Human PCK1, Insig1, Insig1 Δloop1, Insig2, and Insig2 Δloop1 were amplified by PCR and cloned into the pLenti/puro (+) ‐3Flag and pGEX‐4T‐1 (GST) vectors. The Insig1 Δloop1, Insig2 Δloop1 truncations, Flag‐Insig1 S207A, and Flag‐Insig1 S207D were generated using the QuikChange site‐directed mutagenesis kit (Stratagene, La Jolla, CA).

The shRNA sequences used in this study were as follows: control shRNA, GAATCGTCGTATGCAGTGAAA; *ITGB3* shRNA, GATGCAGTGAATTGTACCTAT; *ITGAV* shRNA, GACTGAGCTAATCTTGAGAAT; *Insig1* shRNA, TAATGGTGTCTATCAGTATAC

### Cell Lines and Cell Culture

Huh7 cells were obtained from the American Type Culture Collection (ATCC), and Hep3B cells and HCT116 cells were acquired from the Cell Bank of the Chinese Academy of Sciences. Both cell lines were cultured in DMEM supplemented with 10% fetal bovine serum (FBS; Sigma) and 1% penicillin‐streptomycin (Invitrogen, Thermo Fisher Scientific). The cell lines used in this study were verified to be absent from the International Cell Line Authentication Committee (ICLAC) and NCBI Biosample databases of commonly misidentified cell lines. All cells were routinely tested for mycoplasma contamination and authenticated by short tandem repeat (STR) profiling. For IGF1 treatment, cells were serum‐starved for 16 h prior to stimulation with 100 ng mL^−1^ recombinant IGF1. For transfection experiments, cells were seeded at densities of 4 × 10⁵ cells per 60‐mm dish or 1 × 10⁵ cells per well in a 6‐well plate 18 h before transfection. Transfections were performed using either Lipo8000 Transfection Reagent (Beyotime) or polyethylenimine (PEI; Polysciences Inc.), following the manufacturers’ protocols.

### Immunoprecipitation and Immunoblotting Analysis

Cellular proteins were extracted using ice‐cold lysis buffer [50 mm Tris‐HCl (pH 7.5), 150 mm NaCl, 0.1% SDS, 1% Triton X‐100, 1 mm DTT, 0.5 mm EDTA] supplemented with protease inhibitor cocktail (Roche) and phosphatase inhibitors (Bimake). Following lysis, cell extracts were centrifuged at 13400 × g for 15 min at 4 °C to remove cellular debris. The clarified supernatants were then incubated with primary antibodies overnight at 4 °C with gentle rotation. For Flag‐tagged protein immunoprecipitation, Flag M2 magnetic beads (Sigma) were added to the lysate‐antibody mixture and incubated for an additional 4 h at 4 °C. The immunocomplexes were washed three times with lysis buffer under stringent conditions (5 min per wash with rotation). Finally, the precipitated proteins were eluted and analyzed by immunoblotting as previously described.^[^ ^35^ ^]^

### Purification of Recombinant Proteins

Wild‐type GST‐PCK1 and GST‐PCK1 pS90 proteins were expressed in Escherichia coli and purified as previously described.^[^ ^36^ ^]^ For GST‐PCK1, the full‐length human PCK1 coding sequence was cloned into the pGEX‐4T‐1 expression vector and transformed into BL21(DE3) competent cells. For the GST‐ PCK1 pS90 recombinant protein, the codon at the S90 site of PCK1 is mutated to a stop codon (TCA to TAG). The modified coding sequence was subsequently cloned into the pGEX‐4T‐1 vector and expressed in the engineered competent cells.^[^ ^37^ ^]^ Protein expression was induced in bacterial cultures grown at 37 °C to an OD600 of 0.6 by adding 0.5 mm isopropyl β‐D‐1‐thiogalactopyranoside (IPTG), followed by overnight incubation at 25 °C with shaking at 200 rpm. Bacterial cells were harvested by centrifugation at 4000 × g for 15 min at 4 °C.

For protein purification, cell pellets were resuspended in GST lysis buffer (50 mm Tris‐HCl, pH 8.0, 150 mm NaCl, 1 mM DTT) containing protease inhibitors and lysed by sonication. The clarified lysates were incubated with glutathione‐Sepharose 4B beads (Sangon Biotech) for 4 h at 4 °C with gentle rotation. After extensive washing with five column volumes of GST lysis buffer, bound proteins were eluted using 10 mm reduced glutathione.

### Bio‐Layer Interferometry (BLI) Assay

Biotinylated GST‐PCK1 protein and GST‐PCK1 pS90 protein were prepared, and the BLI analysis was performed as previously described.^[^ ^38^ ^]^ Briefly, the protein was dissolved in PBS and subjected to biotinylate with G‐MM‐IGT Biotinylation kit (Genemore). Before each assay, streptavidin (SA) biosensors were pre‐wetted in PBS to record the baseline. The biotinylated human GST‐PCK1 or GST‐PCK1 pS90 protein was immobilized on SA‐coated 96‐well plate surface, after which a 200 µL solution containing various concentrations of Insig1/2 loop 1 peptide diluted by PBS (containing 0.02% Tween 20) was added to each well. The binding experiment was performed with repeated cycles consisting of four major steps: initial loading, baseline, association, and dissociation. Data collection and analysis were performed by ForteBio Octet R8 (Sartorius).

### Immunofluorescence Analysis

For endogenous SREBP1 and SREBP2 staining, the cells were washed with ice‐cold PBS and fixed with 4% paraformaldehyde in PBS at room temperature (around 20–25 °C) for 15 min. Then, the cells were treated with 0.1% Triton X‐100 for 5 min and blocked in 3% BSA for 1 h at room temperature. The cells were then incubated with primary antibody against SREBP1 (diluted 1:200) or SREBP2 (diluted 1:200) overnight at 4 °C. After incubation with fluorescent‐dye‐conjugated secondary antibody (diluted 1:1000; Thermo Fisher Scientific) in PBS containing 0.05% saponin, 5% goat serum and 0.5% BSA for 1 h at room temperature followed by DAPI (diluted 1:10 000; Thermo Fisher Scientific) staining of the cell nucleus for 10 min. Cells were examined using a laser confocal microscope (Zeiss LSM 900).

### SRE Luciferase Assay

SRE luciferase activity in cell lysates was measured with the luciferase assay system as previously described.^[^ ^15^ ^]^ In brief, Huh7 cells in 24‐well plates were transfected with 0.1 µg SRE‐driven luciferase reporter and 0.075 µg β‐galactosidase. At 24 h after the transfection, the cells were subjected to different culture media. Cell lysates were measured for the activity of luciferase and β‐galactosidase on the basis of the manufacturer's instruction (Promega).

### Quantitative PCR

Total RNA was extracted from cells and tissue samples using TRIzol reagent according to the manufacturer's instructions (Invitrogen). Equal amounts of RNA samples were used for cDNA synthesis with a TaqMan Reverse Transcription Reagents kit (Applied Biosystems). Quantitative PCR analysis was carried out using a 7500 Real‐Time PCR system (Applied Biosystems) with a SYBR Premix Ex Taq kit (Takara Bio). The following primers were used for quantitative PCR: *FASN*, 5′‐CACAGGGACAACCTGGAGTT‐3′ and 5′‐ACTCCACAGGTGGGAACAAG‐3′; *GPAM*, 5′‐TTGTGGCTTGCCTGCTCCTCTA‐3′ and 5′‐AATCACGAGCCAGGACTTCCTC‐3′; *HMGCS1*, 5′‐GATGTGGGAATTGTTGCCCTT‐3′ and 5′‐ATTGTCTCTGTTCCAACTTCCAG‐3′; *LDLR*, 5′‐AACGGTCATTCACCCAGGTC‐3′ and 5′‐GGCTGAAGAATAGGAGTTGCC‐3′; *ACTIN*, 5′‐ACAATGTGGCCGAGGACTTTGA‐3′ and 5′‐TGTGTGGACTTGGGAGAGGACT‐3′.

### Triglyceride (TG) Detection Assay

Cells were harvested and processed for triglyceride (TG) quantification using the Amplex Red Triglyceride Assay Kit (Beyotime, Catalog No. S0129S) according to the manufacturer's instructions. Briefly, collect 1 million cells and add 200 µL of isopropanol. Centrifuge at 12 000 × g for 3–5 min at 4 °C, and collect the supernatant for subsequent analysis. Dilute the supernatant tenfold with assay buffer, and then add 50 µL of the diluted sample to a 96‐well black plate, add 2 µL of lipase, and mix thoroughly. Incubate at 37 °C for 20 min. Then, add 50 µL of the triglyceride detection working solution and mix well, incubate at 37 °C for 60 min in the dark. Set the excitation wavelength to 560 nm and the emission wavelength to 590 nm to measure the fluorescence intensity.

### Cell Proliferation Assay

Cells were seeded in 96‐well plates at a density of 5000 cells per well in 100 µL complete growth medium. Following a 24‐hour incubation period (37 °C, 5% CO_2_) to permit cell adherence, the culture medium was replaced with fresh low serum contained complete medium containing either Insig1/2 loop1 peptide (0–100 µm concentration range) or PBS. Cells were maintained under these conditions for 72 hours. After treatment, the culture medium was carefully aspirated, and cells were washed twice with 200 µL phosphate‐buffered saline (PBS, pH 7.4). Fixed was performed using 4% paraformaldehyde (100 µL/well) for 15 min at room temperature, followed by three PBS washes (200 µL/well) to remove residual fixative. 0.1% (w/v) crystal violet staining solution was prepared by dissolving crystal violet powder in 10% ethanol, followed by filtration through a 0.22 µm membrane. Each well received 80 µL of staining solution and was incubated for 15 min at room temperature, protected from light. Following staining, the dye solution was removed, and the plates were gently rinsed under running deionized water until the background was clear. After complete air drying at room temperature (minimum 30 min), bound dye was solubilized by adding 80 µL of 33% acetic acid (v/v in distilled water) per well. Plates were agitated at 200 rpm for 10 min to ensure complete dissolution before measuring absorbance at 582 nm using a microplate reader. Background correction was performed using wells containing only acetic acid solution.

### Preparation of Liposome with Different Sizes

Ten milliliters of trichloromethane containing 168 mg of lecithin, 56 mg of cholesterol, and 56 mg of DSPE‐PEG was added to a round‐bottom flask. This solution was then evaporated to form a lipid film. Subsequently, 7 mL of PBS was introduced into the flask, and the mixture was sonicated in an ice‐water bath for 20 min to facilitate liposome formation. To achieve liposomes of distinct sizes, the suspension was extruded through a series of filter membranes with pore sizes of 200 nm, 100 nm, and 50 nm using a liposome extruder.

### Preparation of Liposome with Different Compositions

Initially, a varying mass of C16‐cRGD was dissolved in a 5 mL mixture of trifluoroacetic acid and acetonitrile (v/v, 1:9) and then evaporated using a rotary evaporator to form a film. In addition, 168 mg of lecithin, 56 mg of cholesterol, and 56 mg of DSPE‐PEG were dissolved in 10 mL of trichloromethane and evaporated to create a second lipid film within the same round‐bottom flask. Following this, 7 mL of PBS containing a varying mass of C16‐TAT was added to the flask. The mixture was then sonicated and extruded to prepare liposome. The specific feeding ratios for the components are detailed below.

**Table:** 

Abbreviation	Mass of C16‐cRGD [mg]	Mass of C16‐TAT [mg]	Mass ratio of lecithin: cholesterol: DSPE‐PEG: C16‐cRGD: C16‐TAT: peptide
Lipo‐0	0	0	60: 20: 20: 0: 0
Lipo‐1	56	0	60: 20: 20: 20: 0
Lipo‐2	14	42	60: 20: 20: 5: 15
Lipo‐3	28	28	60: 20: 20: 10: 10
Lipo‐4	42	14	60: 20: 20: 15: 5
Lipo‐5	0	56	60: 20: 20: 0: 20

### Preparation of LNP@Insig1/2 Loop 1 Peptide

Five milliliters mixture of Trifluoroacetic acid and acetonitrile (v/v, 1:9) containing 28 mg of C16‐cRGD was evaporated using a rotary evaporator to form a film. Subsequently, 168 mg of lecithin, 56 mg of cholesterol, and 56 mg of DSPE‐PEG were dissolved in 10 mL of trichloromethane, and this solution was evaporated to form a second film within the same round‐bottom flask. Following this, 7 mL of PBS containing a varying mass of C16‐TAT and 28 mg of a functional peptide was added to the flask. After sonication and extrusion, the pure nanomedicine named LNP@Insig1/2 loop 1 peptide was obtained through dialysis with a molecular weight cut‐off (MWCO) of 300 KD.

### In Vivo Pharmacokinetic Study

The pharmacokinetic assay of Insig1/2 loop 1 peptide and LNP@ Insig1/2 loop 1 peptide was performed in ICR male mice. The male mice were administered intravenously (iv) at a dose of 10 mg kg^−1^. Then, blood samples were collected into tubes containing EDTA at 0.083, 0.25, 0.5, 1, 2, 4, 8, 24 h (iv) after dosing. After centrifugation at 6000 rpm for 10 min at 4 °C, the obtained plasma samples were stored at ‐20 °C. Subsequently, the plasma samples were analyzed by liquid (LC‐MS/MS) chromatography‐tandem mass spectrometry analysis.

### In Vivo Biodistribution and Tumor Accumulation

One million Huh7 cells subcutaneously injected into 5‐week‐old male BALB/c athymic nude mice. When the tumor volume reached ≈300 mm^3^, the nude mice were randomly divided into different groups (n = 3). The mice were fasted for 12 h but allowed free access to water, and were then intravenously injected with LNP@ Insig1/2 loop 1 peptide at a dose of 10 mg kg^−1^. Non‐invasive photography of the mice was performed at different times post‐administration (8, 12, 24, and 48 h) using an IVIS Spectrum Imaging System (PerkinElmer, Inc., MA, USA). At 48 h post‐administration, the mice were euthanized and their tumors, as well as six major organs (brain, heart, liver, spleen, lung, and kidney), were excised for ex vivo imaging. The region‐of‐interest (ROI) analysis using Living Image software (PerkinElmer, Inc., MA, USA) was used to quantify the average fluorescence intensity of signal.

### Characterization of Encapsulation (EE) and Loading Efficiency (LE)

100 µL of purified LNP@ Insig1/2 loop 1 peptide was added to 400 µL acetonitrile contain 1% trifluoroacetic acid. Then, the concentration of peptide was detected by High‐performance liquid chromatography (HPLC). Separation of the peptide was carried out using a reverse phase C18 column (5 µm, 4.6 × 250 mm) with a mobile phase of aqueous solution containing 0.1% trifluoroacetic acid and acetonitrile contain 1% trifluoroacetic acid in the ratio of 50:50 v/v at 220 nm. The flow rate was 1.0 mL min^−1^. The injection volume was 10 µL while the column temperature was maintained at 25 °C.

EE and LE were calculated by following equation:

(1) $$EE=\frac{m_{load}}{m_{total}}$$

(2) $$LE=\frac{m_{load}}{m_{load}+m_{LNPs}}$$

where *m_load_* was mass of loaded Insig1/2 loop 1 peptide, *m_total_* was mass of total mass of peptide, and *m_LNPs_* was mass of LNPs.

### Animal Studies

Male athymic BALB/c nude mice (6 weeks old) sourced from GemPharmatech were used for the in vivo tumor growth assay. The animal studies were conducted as previously described48. The mice were accommodated in specific‐pathogen‐free conditions with a 14 h light and 10 h dark cycle, maintaining an ambient temperature of 21–23 °C and 50% humidity, and with ad libitum access to water and food (Jiangsu Xietong Pharmaceutical Bio‐engineering, 1010019). Although the mice used in this study were male mice, sex was not considered as a factor in the study design, and the findings are not limited to a specific sex. Animal studies were approved by the Research Ethics Committee and complied with the ethical regulations of The First Affiliated Hospital of Zhejiang University School of Medicine (2024‐1734). A total of 1.5 × 10^6^ wild‐type Huh7 cells reconstituted were collected in 20 µL DMEM with 33% matrigel and intrahepatically injected into the livers of 6‐week‐old male BALB/c athymic nude mice (or in 100 µL DMEM and subcutaneously injected into the mice). Six or seven mice constituted a group in each experiment. Mice from each group were treated with different drugs or inhibitors starting 7 days after intrahepatically or subcutaneously injection of tumor cells. Animals were euthanized 28 days after injection. The tumor of each mouse was dissected and then fixed in 4% formaldehyde and embedded in paraffin. The tumor volume was calculated using the formula: V = 1/2a^2^b (V, volume; a, shortest diameter; b, longest diameter). The animals were treated in accordance with relevant institutional and national guidelines and regulations. Animals were randomly allocated to experimental groups. No statistical methods were used to predetermine sample size.

### Immunohistochemical (IHC) Analysis

As described previously,^[^ ^39^ ^]^ IHC staining was performed using the Vectastain ABC kit (Vector Laboratories) according to the manufacturer's instructions. Sections of paraffin‐embedded xenograft tumors were stained with antibodies against Ki67, SREBP1, Insig1 pS207/Insig2 pS151, and nonspecific IgG was used as a negative control. Paraffin‐embedded human liver tumor sections were stained with antibodies against Ki67, SREBP1, Insig1 pS207/Insig2 pS151, with nonspecific IgG as a negative control. The tissue sections were quantitatively scored according to the percentage of positive cells and staining intensity. The following proportion scores were assigned: 0, if 0% of the tumor cells showed positive staining; 1, if 0.1% to 1% of cells showed positive staining; 2, if 1.1% to 10% showed positive staining; 3, if 11% to 30% showed positive staining; 4, if 31% to 70% showed positive staining; and 5, if 71% to 100% showed positive staining. The intensity of staining was rated on a scale of 0 to 3: 0, negative; 1, weak; 2, moderate; and 3, strong. The study then combined the proportion and intensity scores to obtain a total score (range, 0–8).

### TUNEL Assay

Mouse tumor tissues were sectioned at 5 µm thickness. Apoptotic cells were counted using the colorimetric TUNEL System (Beyotime, C1091) according to the manufacturer's instructions. The total numbers of apoptotic cells were counted in six high‐power fields and presented as an average number of apoptotic cells per high‐power fields.

### Oil Red O Staining

Cryosections 6–8 µm in thickness were air‐dried and fixed with formaldehyde for 5–10 min. Then, the slides were placed in absolute propylene glycol for 2–5 min and stained with 0.5% Oil red O dye. After staining, the slides were rinsed, counterstained with Mayer's hematoxylin for 1 min, and mounted in glycerin.

### Lipid Uptake Assay

Cells were seeded in 8‐well chambered coverslip (Cellvis) and cultured for 24 h (37 °C, 5% CO_2_), followed by optional serum starvation in serum‐free medium for 4 h. BODIPY FL C16 was dissolved in DMSO to 1 mm stock and diluted to 10 µm in serum‐free medium. Cells were pretreated with semaglutide for 1 h, then incubated with BODIPY FL C16 solution for 30 min at 37 °C, washed three times with ice‐cold PBS, and lysed with 1% Triton X‐100. DAPI (diluted 1:10 000; Thermo Fisher Scientific) staining of the cell nucleus for 10 min. Cells were examined using a laser confocal microscope (Zeiss LSM 900).

### Quantification and Statistical Analysis

All quantitative data are presented as the mean ± standard deviation (SD) of at least three independent experiments. A two‐group comparison was conducted using the unpaired two‐tailed Student's *t* test. Multiple groups and/or multiple condition comparisons were conducted using one‐way or two‐way analysis of variance (ANOVA). Overall survival was analyzed with Kaplan‐Meier curves, and differences in survival among subgroups were analyzed using the log‐rank test. Values of *p* < 0.05 were considered statistically significant. Statistical analysis was carried out using GraphPad Prism software for statistical significance.

## Conflict of Interest

The authors declare no conflict of interest.

## Author Contributions

S.L., H.Y., and X.J. contributed equally to this work. Z.L. conceptualized the study. Z.L., Z.M., S.L., D.X., and H.Y. designed the study and wrote the manuscript. Z.M. and Z.L. acquired funding support and supervised the study. S.L., H.Y., X.J., X.H., Z.W., M.L., S.L., and Y.M. performed the experiments and statistical analysis. M.L. and S.L. reviewed the manuscript and provided technical support.

## Supporting information



Supporting Information

## Data Availability

The data that support the findings of this study are available from the corresponding author upon reasonable request.

## References

[1] J. L. Goldstein , R. A. DeBose‐Boyd , M. S. Brown , Cell 2006, 124, 35. 16413480 10.1016/j.cell.2005.12.022

[2] F. Röhrig , A. Schulze , Nat. Rev. Cancer 2016, 16, 732. 27658529 10.1038/nrc.2016.89

[3] H. Shimano , R. Sato , Nat. Rev. Endocrinol. 2017, 13, 710. 28849786 10.1038/nrendo.2017.91

[4] A. Nohturfft , D. Yabe , J. L. Goldstein , M. S. Brown , P. J. Espenshade , Cell 2000, 102, 315. 10975522 10.1016/s0092-8674(00)00037-4

[5] M. S. Brown , J. L. Goldstein , Cell 1997, 89, 331.9150132

[6] J. D. Feramisco , J. L. Goldstein , M. S. Brown , J. Biol. Chem. 2004, 279, 8487. 14660594 10.1074/jbc.M312623200

[7] D. Yabe , M. S. Brown , J. L. Goldstein , Proc. Natl. Acad. Sci. USA 2002, 99, 12753. 12242332 10.1073/pnas.162488899 PMC130532

[8] R. Ren , X. Zhou , Y. He , M. Ke , J. Wu , X. Liu , C. Yan , Y. Wu , X. Gong , X. Lei , S. F. Yan , A. Radhakrishnan , N. Yan , Science 2015, 349, 187. 26160948 10.1126/science.aab1091 PMC4704858

[9] A. Radhakrishnan , Y. Ikeda , H. J. Kwon , M. S. Brown , J. L. Goldstein , Proc. Natl. Acad. Sci. USA 2007, 104, 6511. 17428920 10.1073/pnas.0700899104 PMC1851665

[10] Y. Gong , J. N. Lee , M. S. Brown , J. L. Goldstein , J. Ye , Proc. Natl. Acad. Sci. USA 2006, 103, 6195. 10.1073/pnas.0601923103 PMC1435366 16606821

[11] L.‐P. Sun , J. Seemann , J. L. Goldstein , M. S. Brown , Proc. Natl. Acad. Sci. USA 2007, 104, 6519. 17428919 10.1073/pnas.0700907104 PMC1851663

[12] P. J. Espenshade , J. Cell Sci. 2006, 119, 973. 16525117 10.1242/jcs.02866

[13] L.‐P. Sun , L. Li , J. L. Goldstein , M. S. Brown , J. Biol. Chem. 2005, 280, 26483. 15899885 10.1074/jbc.M504041200

[14] C. Hammond , A. Helenius , J. Cell Biol. 1994, 126, 41. 8027184 10.1083/jcb.126.1.41 PMC2120101

[15] D. Xu , Z. Wang , Y. Xia , F. Shao , W. Xia , Y. Wei , X. Li , X. Qian , J.‐H. Lee , L. Du , Y. Zheng , G. Lv , J.‐S. Leu , H. Wang , D. Xing , T. Liang , M.‐C. Hung , Z. Lu , Nature 2020, 580, 530. 32322062 10.1038/s41586-020-2183-2

[16] H. Jiang , L. Zhu , D. Xu , Z. Lu , Cancer Commun. 2020, 40, 389. 10.1002/cac2.12084 PMC7494067 32809272

[17] M. Li , Z. Wang , J. Tao , H. Jiang , H. Yang , D. Guo , H. Zhao , X. He , S. Luo , X. Jiang , L. Yuan , L. Xiao , H. He , R. Yu , J. Fang , T. Liang , Z. Mao , D. Xu , Z. Lu , Nat. Chem. Biol. 2024, 20, 1505. 38538923 10.1038/s41589-024-01597-2

[18] X. Hou , T. Zaks , R. Langer , Y. Dong , Nat. Rev. Mater. 2021, 6, 1078. 34394960 10.1038/s41578-021-00358-0 PMC8353930

[19] E. Kon , N. Ad‐El , I. Hazan‐Halevy , L. Stotsky‐Oterin , D. Peer , Nat. Rev. Clin. Oncol. 2023, 20, 739. 37587254 10.1038/s41571-023-00811-9

[20] L. J. Kubiatowicz , A. Mohapatra , N. Krishnan , R. H. Fang , L. Zhang , Exploration 2022, 2, 20210217. 36249890 10.1002/EXP.20210217 PMC9539018

[21] Y. Zhao , Y.‐N. Zhang , K.‐T. Wang , L. Chen , Biochim. Biophys. Acta Rev. Cancer 2020, 1874, 188391. 32659252 10.1016/j.bbcan.2020.188391

[22] B. Hu , T. Zou , W. Qin , X. Shen , Y. Su , J. Li , Y. Chen , Z. Zhang , H. Sun , Y. Zheng , C.‐Q. Wang , Z. Wang , T.‐E. Li , S. Wang , L. Zhu , X. Wang , Y. Fu , X. Ren , Q. Dong , L.‐X. Qin , Cancer Res. 2022, 82, 3845. 36066408 10.1158/0008-5472.CAN-21-4140 PMC9574378

[23] Y.‐C. Liu , B.‐H. Huang , J.‐G. Chung , W.‐L. Liu , F.‐T. Hsu , S.‐S. Lin , Anticancer Res. 2021, 41, 123. 33419805 10.21873/anticanres.14757

[24] X. Bian , R. Liu , Y. Meng , D. Xing , D. Xu , Z. Lu , J. Exp. Med. 2021, 218, 20201606. 10.1084/jem.20201606 PMC7754673 33601415

[25] J. Wang , Y. Yang , F. Shao , Y. Meng , D. Guo , J. He , Z. Lu , Nat. Metab. 2024, 6, 914. 38702440 10.1038/s42255-024-01037-4

[26] L. Maretty , D. Gill , L. Simonsen , K. Soh , L. Zagkos , M. Galanakis , J. Sibbesen , M. T. Iglesias , A. Secher , D. Valkenborg , J. Q. Purnell , L. B. Knudsen , A. A. Tahrani , M. Geybels , Nat. Med. 2025, 31, 267. 39753963 10.1038/s41591-024-03355-2 PMC11750704

[27] L. Bu , Z. Zhang , J. Chen , Y. Fan , J. Guo , Y. Su , H. Wang , X. Zhang , X. Wu , Q. Jiang , B. Gao , L. Wang , K. Hu , X. Zhang , W. Xie , W. Wei , M. Kuang , J. Guo , Gut 2024, 73, 1156. 38191266 10.1136/gutjnl-2023-330826

[28] Y. He , S. Zhang , Y. She , Z. Liu , Y. Zhu , Q. Cheng , X. Ji , Exploration 2024, 4, 20230164. 39713200 10.1002/EXP.20230164 PMC11655310

[29] M. Dymek , E. Sikora , Adv. Colloid Interface Sci. 2022, 309, 102757. 36152374 10.1016/j.cis.2022.102757

[30] X. Ma , T. Zhao , H. Yan , K. Guo , Z. Liu , L. Wei , W. Lu , C. Qiu , J. Jiang , Cell Death Dis. 2021, 12, 544. 34039951 10.1038/s41419-021-03762-0 PMC8155186

[31] T.‐B. Wang , M. Geng , H. Jin , A.‐G. Tang , H. Sun , L.‐Z. Zhou , B.‐H. Chen , G. Shen , Q. Sun , Cell Death Dis. 2021, 12, 717. 34285190 10.1038/s41419-021-03999-9 PMC8292369

[32] S. H. Lee , J.‐H. Lee , S.‐S. Im , Exp. Mol. Med. 2020, 52, 724. 32385422 10.1038/s12276-020-0430-0 PMC7272406

[33] Q. Ren , S. Chen , X. Chen , S. Niu , L. Yue , X. Pan , Z. Li , X. Chen , Drug Des. Dev. Ther. 2022, 16, 3723. 10.2147/DDDT.S381546 PMC9594960 36304787

[34] N. C. Bergmann , M. J. Davies , I. Lingvay , F. K. Knop , Diabetes, Obes. Metab. 2022, 25, 18. 36254579 10.1111/dom.14863 PMC10092086

[35] Z. Lu , D. Liu , A. Hornia , W. Devonish , M. Pagano , D. A. Foster , Mol. Cell. Biol. 1997, 18, 839. 10.1128/mcb.18.2.839 PMC108795 9447980

[36] R. Liu , J.‐H. Lee , J. Li , R. Yu , L. Tan , Y. Xia , Y. Zheng , X.‐L. Bian , P. L. Lorenzi , Q. Chen , Z. Lu , Mol. Cell 2021, 81, 2722. 34077757 10.1016/j.molcel.2021.05.005

[37] X. Liu , W. Xiao , Y. Zhang , S. E. Wiley , T. Zuo , Y. Zheng , N. Chen , L. Chen , X. Wang , Y. Zheng , L. Huang , S. Lin , A. N. Murphy , J. E. Dixon , P. Xu , X. Guo , Proc. Natl. Acad. Sci. USA 2019, 117, 328.31843888

[38] J. Wallner , G. Lhota , D. Jeschek , A. Mader , K. Vorauer‐Uhl , J. Pharm. Biomed. Anal. 2013, 72, 150. 23146240 10.1016/j.jpba.2012.10.008

[39] X. Qian , X. Li , Z. Shi , Y. Xia , Q. Cai , D. Xu , L. Tan , L. Du , Y. Zheng , D. Zhao , C. Zhang , P. L. Lorenzi , Y. You , B.‐H. Jiang , T. Jiang , H. Li , Z. Lu , Mol. Cell 2019, 76, 516. 31492635 10.1016/j.molcel.2019.08.006

