# Supplementary material for: Inhibition of Tumor Lipogenesis and Growth by Peptide‐Based Targeting of SREBP Activation
Source: Adv Sci (Weinh). 2025 Sep 17;12(45):e08111. doi: 10.1002/advs.202508111 (PMC12677653; doi:10.1002/advs.202508111)
Supplement: Supplementary file 1 — Supporting Information [file ADVS-12-e08111-s001.docx]

**Supporting Information**

**Inhibition of tumor lipogenesis and growth by peptide-based targeting of SREBP activation**

Shudi Luo^1, 2#^, Huang Yang^3#^, Xiaoming Jiang^1, 2#^, Zheng Wang^1, 2^, Xuxiao He^1, 2^, Ying Meng^1, 2^, Shan Li^1, 2^, Min Li^1, 2^, Daqian Xu^1, 2*^, Zhengwei Mao^3*^, Zhimin Lu^1, 2*^

^1^Zhejiang Key Laboratory of Pancreatic Disease, Department of Gastroenterology, The First Affiliated Hospital, Zhejiang Key Laboratory of Frontier Medical Research on Cancer Metabolism, Institute of Translational Medicine, Zhejiang University School of Medicine, Hangzhou, Zhejiang 310029, China

^2^ Institute of Fundamental and Transdisciplinary Research, Cancer Center, Zhejiang University, Hangzhou, Zhejiang 310029, China

^3^MOE Key Laboratory of Macromolecular Synthesis and Functionalization, Department of Polymer Science and Engineering, Zhejiang University, Hangzhou 310027, China

*^#^*Equal contribution: These authors contribute equally to this work.

^*^**Correspondence:** zhiminlu@zju.edu.cn (Z. L.), zwmao@zju.edu.cn (Z. M.), xudaqian@zju.edu.cn (D. X).

**Figure S1. The Insig1/2 loop 1 peptide binds to S90-phosphorylated PCK1 and** **blocks PCK1-mediated SREBP activation and tumor cell proliferation**


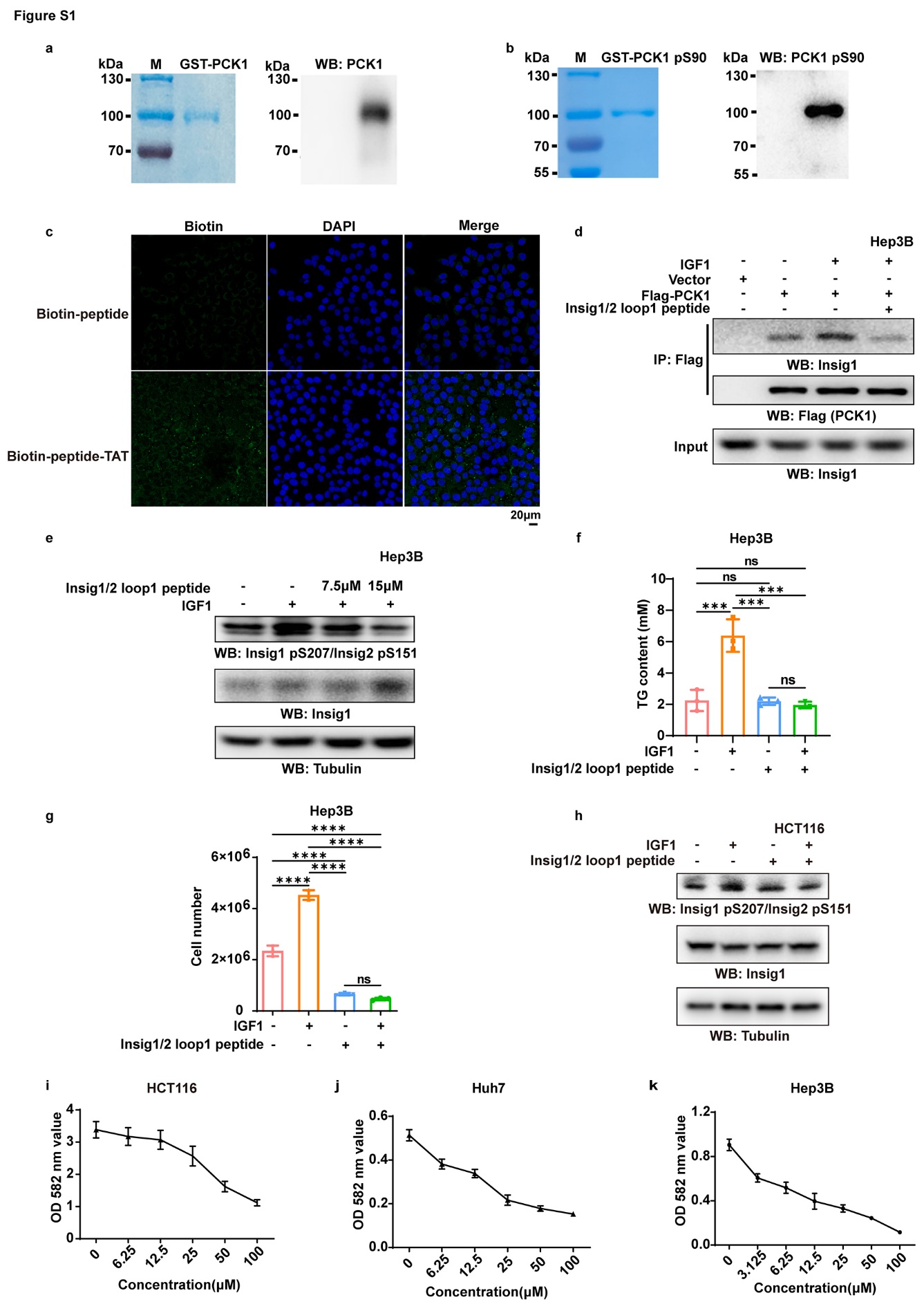


Immunoblotting analyses were performed using the indicated antibodies; representative results from three independent experiments are shown (IP, immunoprecipitation; WB, western blot).

**a, b)** Recombinant protein purification. **(a)** GST-PCK1 and **(b)** GST-PCK1 pS90 proteins purified from E. coli were analyzed by Coomassie brilliant blue staining (left panels) and SDS-PAGE (right panels)

**c)** Huh7 cells were incubated for 30 min with biotin-labeled Insig1/2 loop1 peptide (15 μM), either with or without the TAT sequence, followed by immunofluorescence analysis. (Scale bar: 20 μm)**.**

**d)** Flag-PCK1-expressing Hep3B cells were pre-treated with Insig1/2 loop 1 peptide (15 μM) for 1 h before IGF1 (100 ng ml⁻¹) stimulation.

**e)** Hep3B cells were pretreated with low (7.5 μM) or high (15 μM) concentrations of Insig1/2 loop 1 peptide for 1 h before IGF1 (100 ng ml⁻¹) stimulation.

**f)** Intracellular triglyceride (TG) content was quantified in Hep3B cells pretreated with Insig1/2 loop 1 peptide (25 μM) and stimulated with IGF1 (100 ng ml⁻¹) for 24 h.

**g)** Cell proliferation was assessed by automated cell counting after 72 h of IGF1 stimulation (100 ng ml⁻¹) in Hep3B cells pretreated with Insig1/2 loop 1 peptide (25 μM) for 1 h.

**h)** HCT116 cells were pretreated with Insig1/2 loop1 peptide (15 μM) for 1 h before IGF1 (100 ng ml⁻¹) stimulation.

**i-k)** **(i)** HCT116, **(j)** Huh7, and **(k)** Hep3B cells were exposed to a concentration gradient of Insig1/2 loop1 peptide, and cell proliferation was quantified by optical density (OD) measurements.

Data represent the mean ± SD (n = 3). Statistical significance was determined by one-way analysis of variance (ANOVA). *** p < 0.001, **** p < 0.0001, ns, no significance.

**Figure S2. The Insig1/2 loop 1 peptide inhibits IGF1-mediated SREBP2 activation and suppresses its downstream target gene expression.**


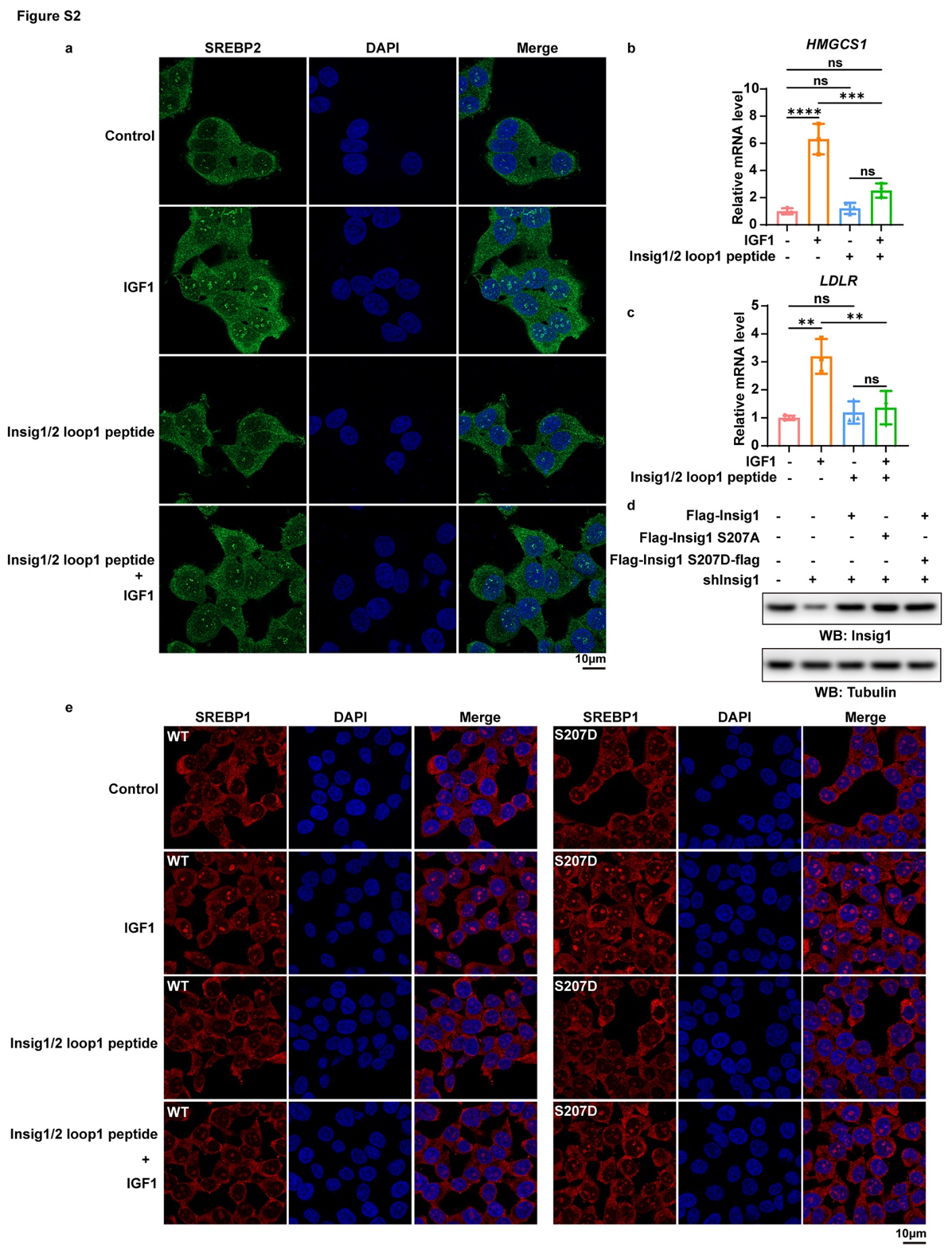


**a)** Following pretreatment with Insig1/2 loop 1 peptide (15 μM) and IGF1 stimulation (100 ng ml⁻¹, 8 h), Huh7 cells were subjected to immunofluorescence analysis (scale bar: 10 μm).

**b, c)** Quantitative PCR analysis of SREBP2 target genes **(b)** *HMGCS1* and **(c)** *LDLR* in Huh7 cells pretreated with Insig1/2 loop 1 peptide (15 μM) and stimulated with IGF1 (100 ng ml⁻¹) for 10 h.

**d)** FLAG-Insig1, Flag-Insig1 S207A or FLAG- Insig1 S207D was expressed in Huh7 cells with depletion of Insig1 by expressing Insig1 shRNA.

**e)** FLAG-Insig1 and FLAG- Insig1 S207D was expressed in Huh7 cells with depletion of Insig1 by expressing Insig1 shRNA. Immunofluorescence analysis of SREBP1 in Huh7 cells pretreated with Insig1/2 loop 1 peptide (15 μM) and stimulated with IGF1 (100 ng ml⁻¹) for 10 h.

Data are expressed as mean ± SD (n = 3). Statistical significance was determined by one-way analysis of variance (ANOVA). ** p < 0.01, *** p < 0.001, **** p < 0.0001, ns, no significance.

**Figure S3. The drug delivery characteristics of LNPs**


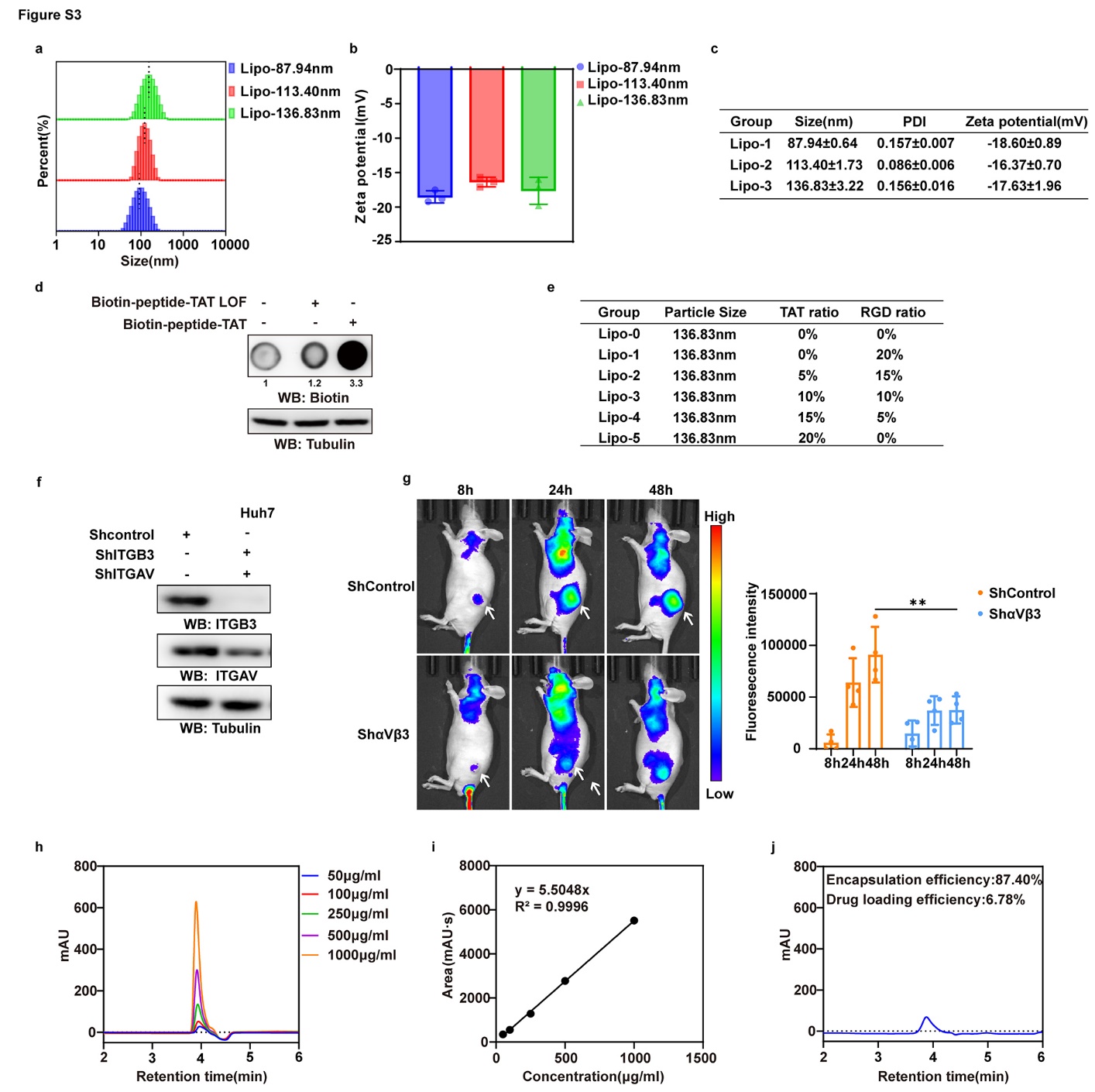


**a-c)** LNPs size optimization and characterization. **(a)** Size distribution, **(b)** surface zeta potential of Lipo-1, Lipo-2, and Lipo-3 and **(c)** Summary table of Lipo-1, Lipo-2, and Lipo-3 in terms of size, polydispersity index (PDI), and zeta potential.

**d)** Huh7 cells were incubated for 30 min with biotin-labeled Insig1/2 loop1 peptide (15 μM), either with TAT or TAT loss of function (TAT-LOF) sequence, with subsequent western blot analysis of cellular peptide uptake.

**e)** The LNPs was modified with varying ratios of TAT and cRGD peptides, designated as Lipo-0, Lipo-1, Lipo-2, Lipo-3, Lipo-4, and Lipo-5.

**f)** Huh7 cells with stable knockdown of ITGB3 and ITGAV achieved through shRNA expression.

**g)** ITGB3/ITGAV-knockdown Huh7 cells were subcutaneously implanted into nude mice (n = 4 per group). Mice were imaged at 8, 24, and 48 h after intravenous administration of RGD-modified LNPs (10% formulation, 10 mg kg⁻¹). Data are expressed as mean ± SD (n = 3). Statistical significance was determined by two-way analysis of variance (ANOVA). ** p < 0.01.

**h)** High-performance liquid chromatography (HPLC) spectrum of different concentrations of peptide solution as indicated.

**i)** Standard curve of peptide solution.

**j)** HPLC spectrum of LNP@Insig1/2 loop 1 peptide solution diluted with acetonitrile containing 1% trifluoroacetic acid (1:4, v/v).

**Figure S4. LNP-mediated delivery of Insig1/2 loop 1 peptide was safe for mice**
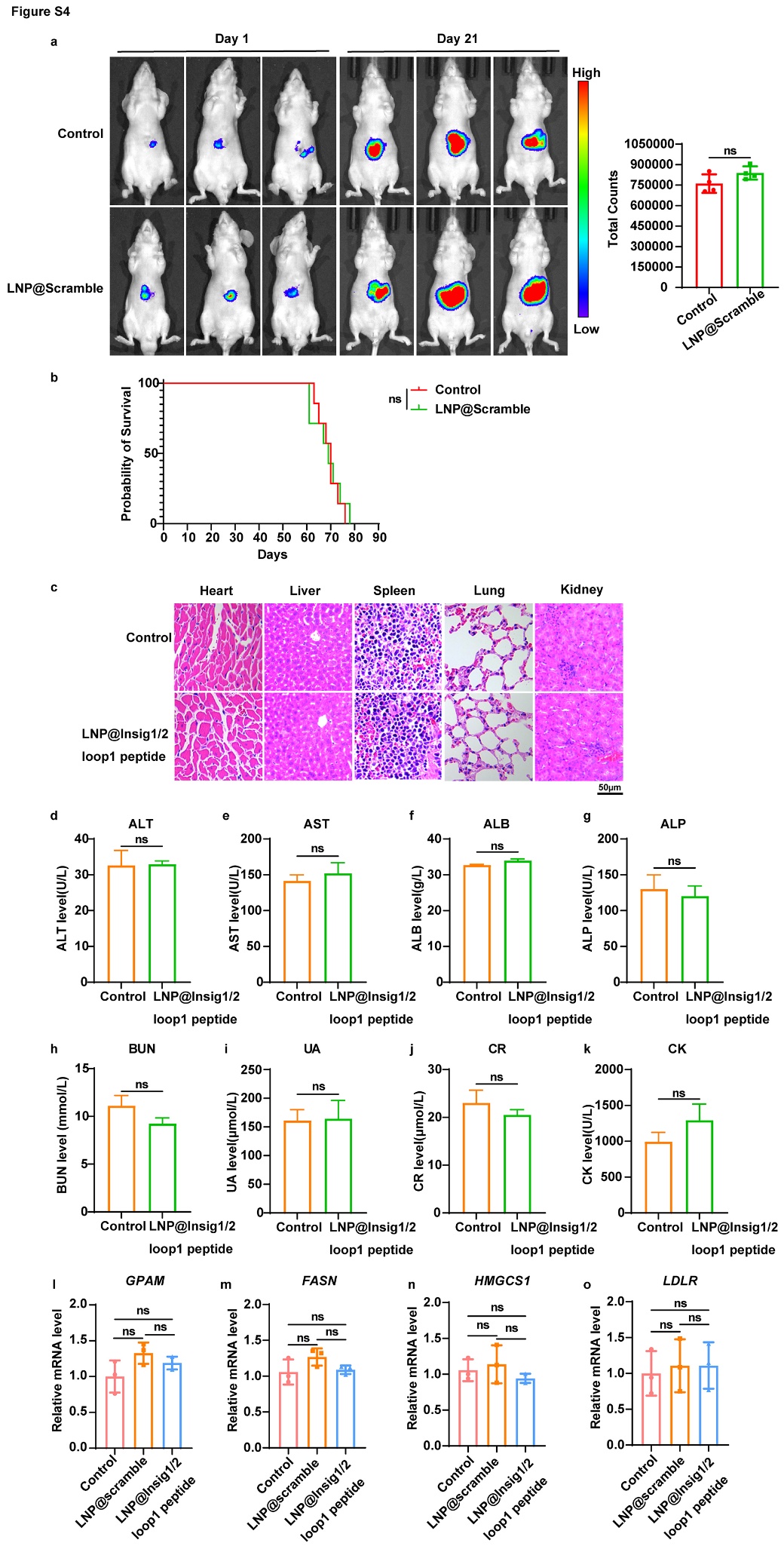


**a,b)** For the orthotopic model, Huh7 cells were intrahepatically injected into nude mice (n = 5 per group). Treatment groups received intravenous injections of LNP@scramble peptide or PBS (10 mg kg⁻¹, every other day). **(a)** Tumor progression was monitored by *in vivo* imaging system. **(b)** Survival analysis was performed using Kaplan-Meier curves. Statistical significance was determined by Log-rank (Mantel-Cox) test.

**c-k)** The LNP@Insig1/2 loop 1 peptide or PBS (control) was administered intravenously to 6-week-old nude mice (n = 4 per group) at a dose of 10 mg kg^-1^ body weight. **(c)**Mice heart, liver, spleen, lung and kidney were harvested for H&E staining (scale bar: 50 μm)**.** Mouse blood was collected for **(d)** ALT, **(e)** AST, **(f)** ALB, **(g)** ALP, **(h)** BUN, **(i)** UA, **(j)** CR, **(k)** CK analysis. ALT, alanine aminotransferase; AST, aspartate transaminase; ALB, albumin; ALP, alkaline phosphatase; BUN, blood urea nitrogen; UA, uric acid; CR, creatinine; CK, creatine kinase. Data are expressed as mean ± SD. Comparisons between the two groups were performed using Student's *t*-test.

**l-o)** The LNP@Insig1/2 loop 1 peptide or scramble peptide were administered intravenously to 6-week-old nude mice (n = 3 per group) at a dose of 10 mg kg^-1^ body weight. Expression levels of lipid metabolism genes **(l)** *GPAM*, **(m)** *FASN*, **(n)** *HMGCS1*, and **(o)** *LDLR* were quantified by quantitative PCR in normal mouse liver tissues. Data are expressed as mean ± SD (n = 3), multiple comparisons were performed using analysis of variance (ANOVA).

ns, no significance.

**Figure S5.** **Semaglutide reduces circulating LDL-C and HDL-C independently of the PCK1-Insig-SREBP axis**


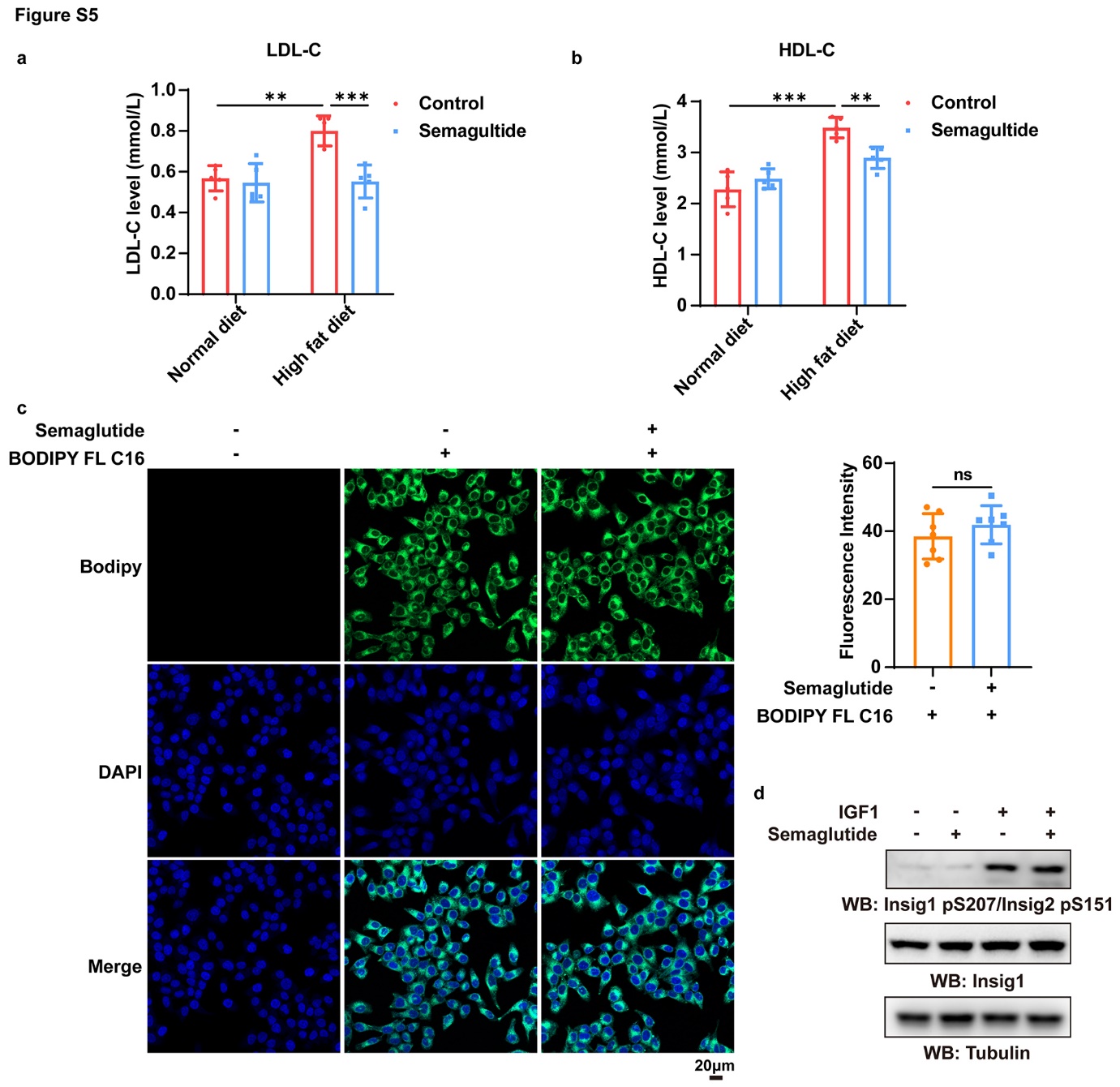


**a, b)** Nude mice (n=5 per group) with orthotopic Huh7 cell liver implants were treated with daily intraperitoneal injections of semaglutide (30 nmol kg⁻¹, daily). Blood samples were collected for analysis of **(a)** LDL-C and **(b)** HDL-C levels. Data are expressed as mean ± SD. Statistical significance was determined by two-way analysis of variance (ANOVA).

**c)** Huh7 cells were pretreated with semaglutide (1μM) for 1 hour prior to incubation with the fluorescent fatty acid analog BODIPY FL C16. Cellular lipid uptake was visualized and quantified using BODIPY immunostaining. Data are expressed as mean ± SD (n = 7), comparisons between the two groups were performed using Student's *t*-test.

**d)** Huh7 cells were pretreated with semaglutide (1μM) for 1 h before IGF1 (100 ng ml⁻¹) stimulation.

** p < 0.01, *** p < 0.001, ns, no significance.
